# Impact of Sit-to-Stand and Treadmill Desks on Patterns of Daily Waking Physical Behaviors Among Overweight and Obese Seated Office Workers: Cluster Randomized Controlled Trial

**DOI:** 10.2196/43018

**Published:** 2023-05-16

**Authors:** Diego Arguello, Gregory Cloutier, Anne N Thorndike, Carmen Castaneda Sceppa, John Griffith, Dinesh John

**Affiliations:** 1 Human Performance and Exercise Science Lab Department of Health Sciences Northeastern University Boston, MA United States; 2 Center for Cognitive and Brain Health College of Science Northeastern University Boston, MA United States; 3 Department of Medicine Massachusetts General Hospital Boston, MA United States; 4 Harvard Medical School Boston, MA United States; 5 Bouve College of Health Sciences Institute on Urban Health Research Northeastern University Boston, MA United States; 6 Department of Health Sciences Northeastern University Boston, MA United States

**Keywords:** prolonged sedentary behavior, sedentary behavior, sit-to-stand desks, treadmill desks, physical activity promotion, workplace wellness, seated office workers, move more and sit less

## Abstract

**Background:**

Sit-to-stand and treadmill desks may help sedentary office workers meet the physical activity guideline to “move more and sit less,” but little is known about their long-term impact on altering the accumulation patterns of physical behaviors.

**Objective:**

This study explores the impact of sit-to-stand and treadmill desks on physical behavior accumulation patterns during a 12-month multicomponent intervention with an intent-to-treat design in overweight and obese seated office workers.

**Methods:**

In total, 66 office workers were cluster randomized into a seated desk control (n=21, 32%; 8 clusters), sit-to-stand desk (n=23, 35%; 9 clusters), or treadmill desk (n=22, 33%; 7 clusters) group. Participants wore an activPAL (PAL Technologies Ltd) accelerometer for 7 days at baseline, 3-month follow-up (M3), 6-month follow-up (M6), and 12-month follow-up (M12) and received periodic feedback on their physical behaviors. Analyses of physical behavior patterns included total day and workday number of sedentary, standing, and stepping bouts categorized into durations ranging from 1 to 60 and >60 minutes and usual sedentary, standing, and stepping bout durations. Intervention trends were analyzed using random-intercept mixed linear models accounting for repeated measures and clustering effects.

**Results:**

The treadmill desk group favored prolonged sedentary bouts (>60 min), whereas the sit-to-stand desk group accrued more short-duration sedentary bouts (<20 min). Therefore, compared with controls, sit-to-stand desk users had shorter usual sedentary bout durations short-term (total day ΔM3: −10.1 min/bout, 95% CI −17.9 to −2.2; *P*=.01; workday ΔM3: −20.3 min/bout, 95% CI −37.7 to −2.9; *P*=.02), whereas treadmill desk users had longer usual sedentary bout durations long-term (total day ΔM12: 9.0 min/bout, 95% CI 1.6-16.4; *P*=.02). The treadmill desk group favored prolonged standing bouts (30-60 min and >60 min), whereas the sit-to-stand desk group accrued more short-duration standing bouts (<20 min). As such, relative to controls, treadmill desk users had longer usual standing bout durations short-term (total day ΔM3: 6.9 min/bout, 95% CI 2.5-11.4; *P*=.002; workday ΔM3: 8.9 min/bout, 95% CI 2.1-15.7; *P*=.01) and sustained this long-term (total day ΔM12: 4.5 min/bout, 95% CI 0.7-8.4; *P*=.02; workday ΔM12: 5.8 min/bout, 95% CI 0.9-10.6; *P*=.02), whereas sit-to-stand desk users showed this trend only in the long-term (total day ΔM12: 4.2 min/bout, 95% CI 0.1-8.3; *P*=.046). The treadmill desk group accumulated more stepping bouts across various bins of duration (5-50 min), primarily at M3. Thus, treadmill desk users had longer usual stepping bout durations in the short-term compared with controls (workday ΔM3: 4.8 min/bout, 95% CI 1.3-8.3; *P*=.007) and in the short- and long-term compared with sit-to-stand desk users (workday ΔM3: 4.7 min/bout, 95% CI 1.6-7.8; *P*=.003; workday ΔM12: 3.0 min/bout, 95% CI 0.1-5.9; *P*=.04).

**Conclusions:**

Sit-to-stand desks exerted potentially more favorable physical behavior accumulation patterns than treadmill desks. Future active workstation trials should consider strategies to promote more frequent long-term movement bouts and dissuade prolonged static postural fixity.

**Trial Registration:**

ClinicalTrials.gov NCT02376504; https://clinicaltrials.gov/ct2/show/NCT02376504

## Introduction

### Background

Sedentary behavior is an independent risk factor for chronic disease [1,2]. For example, excessive and prolonged sedentary behavior, in particular, has an independent effect on all-cause and cardiovascular disease mortality, irrespective of participation in exercise [3-6]. As a result, the 2018 Physical Activity Guidelines for Americans [2] emphasized the promotion of active lifestyles (ie, “move more and sit less” [4]) outside of exercise as an essential component of chronic disease prevention.

The workplace is considered to be a priority setting to reduce sedentary behavior [7] because a substantial proportion of the workforce is employed in full-time seated desk jobs [8-10]. Treadmill and sit-to-stand desks have gained popularity as alternatives to traditional seated workstations to promote interruptions in workplace sitting. A better understanding of how these desks alter the accumulation of physical behaviors among sedentary office workers may be useful to develop effective strategies to “move more and sit less” [4]. Patterns of physical behavior accumulation may be of public health importance, as recent experimental evidence shows that breaking up prolonged sitting with regular activity interruptions (ie, “sit less, move more and more often” [4]) improves lower-limb vascular function [11], blood pressure [12-14], hemodynamics [15], and glucose and insulin regulation [16]. Several nationally representative longitudinal surveillance studies (ie, Australian Diabetes, Obesity, and Lifestyle Study and US National Health and Nutrition Examination Survey) also found protective cardiometabolic health effects when sedentary behavior was frequently replaced with light-intensity activities [17-19]. Furthermore, total activity volume, which captures both light and intermittent moderate to vigorous physical activity, has been shown to have stronger protective associations with various cardiometabolic health biomarkers (ie, lipoproteins, glucose and insulin regulation, inflammatory markers, and measures of adiposity) as compared with exercise [20,21]. However, little experimental evidence exists on the impact of treadmill and sit-to-stand desks on the daily transitional balance between the time spent in static postures (ie, sitting and standing) versus stepping. Such evidence may be useful in tackling the public health challenge of promoting sitting less and moving more often in at-risk populations.

### Prior Work

To date, most treadmill and sit-stand desk studies have reported effects on the overall volume of time spent in waking physical behaviors, with minimal information on the pattern of accumulating behavioral bouts [22-33]. For example, the sitting reduction effects of treadmill desks remain unclear or inconsistent [34] because of the small number of robust studies (eg, [22,24]), but sit-to-stand desks have been shown to effectively reduce workplace sitting volume by an average of 100 minutes per day short-term (≤3 months) and 57 minutes per day on average between follow-up periods of 3 to 12 months [34], with the largest drop off at 12 months follow-up (ie, mean range −37 to −42 min/day) [27,28,35]. However, it is unclear whether active workstations reduce sitting in an all-or-none fashion, similar to that of exercise participation behavior, or whether they produce regular breaks from sitting throughout the day. Current knowledge on how active workstations alter physical behavior patterns consists of only a select number of metrics, such as usual or mean sedentary bout durations, selected duration stratifications of prolonged sedentary bouts (ie, >30 min), the number of sit-to-upright transitions or breaks from sedentary behavior, or the number of daily bouts of various physical behaviors [22,24,27,28,31,36,37]. Previously, we reported that our sit-to-stand and treadmill desk trial resulted in short-term improvements in standing (ie, 62-74 min/day) and stepping volume (ie, 41-49 min/day), with a pattern effect of treadmill desk users engaging in fewer daily sedentary bouts, whereas sit-to-stand desk users transitioned more frequently to upright physical behaviors [22]. As these data were collected using wearable sensors, they can be further examined at a level of granularity, which enables the study of the full spectrum of how sedentary, standing, and stepping bout patterns are altered by desk-based sedentary behavior interventions. To determine whether such interventions are suitable for meeting the 2018 Physical Activity Guidelines’ public health mandate to promote sitting less and moving more often, it may be necessary to report pattern metrics beyond bout duration distributions and measures of central tendency (ie, usual bout durations [28]) for both sedentary and upright behaviors (eg, standing and stepping). This may enable more intervention tailoring, which may not be possible with measures of central tendency.

### Objectives of This Study

This study aimed to improve the understanding of how sit-to-stand and treadmill desks alter the daily accumulation patterns of physical behaviors by exploring broader pattern outcome metrics than those in previous reports. Aim 1 assessed the impact of sit-to-stand and treadmill desks on the daily accumulation of total waking day and workday sedentary, standing, and stepping bouts categorized by bout duration (ie, 1-4.9 min, 5-9.9 min, 10-19.9 min, 20-29.9 min, 30-39.9 min, 40-49.9 min, 50-59.9 min, and >60 min). This aim expands on previous reports [24,27,28,31,36,37] of limited physical behavior bout bin classifications, such as >30 minutes of unbroken sedentary time, which were based on evidence suggesting that such durations may confer greater cardiometabolic risk than sitting for shorter periods [3-6,16,18], and also includes distributions of standing and stepping bouts. Aim 2 assessed the impact of sit-to-stand and treadmill desks on the usual [28,38] sedentary, standing, and stepping bout durations over the total waking day and workday. The latter 2 outcomes have not been previously reported. Bins of physical behavior bouts at the resolution described in aim 1 may explain within- and between-group changes in usual physical behavior bout durations of aim 2.

## Methods

### Overview

The data analyzed in this study were from a 12-month, 3-arm cluster randomized controlled trial titled “Modifying the Workplace to Decrease Sedentary Behavior and Improve Health” (ClinicalTrials.gov NCT02376504), which was conducted between March 2014 and December 2016. The full protocol detailing all the measures used in the study as well as enrollment and attrition figures are available in Multimedia Appendices 1-3. We provide the brief details relevant to this study in the subsequent sections.

### Ethics Approval

Ethics approval (#2013P001695) was granted by the institutional review boards of Partners HealthCare and Northeastern University in March 2014, and all participants provided written informed consent upon enrollment.

### Setting and Participants

A total of 66 office workers were recruited in clusters from the Massachusetts General Hospital and Northeastern University in Boston, Massachusetts (ie, Massachusetts General Hospital: 19 clusters; n=60, 91%; Northeastern University: 5 clusters; n=6, 9%). These clusters were randomized into the following groups: seated desk control (21/66, 32%; 8 clusters), sit-to-stand desk (23/66, 35%; 9 clusters), and treadmill desk intervention (22/66, 33%; 7 clusters) groups. Participant clusters were identified based on office space, such that clusters were separated by walls or were located on a different floor or building. Separations were aimed at preventing participants in one cluster from being visible to other clusters during day-to-day office activities. Employees within these clusters were considered eligible if they were employed in a full-time seated desk job, were aged between 18 and 65 years, had a BMI of >25 kg/m^2^, did not engage in any structured physical activity for >2 days per week, and were free of limitations that prevented walking and standing in bouts lasting 40 to 60 minutes. Participants were screened for hypertension, diabetes, cardiovascular disease, and musculoskeletal conditions (ie, joint, bone, or muscle conditions) using a medical history questionnaire at baseline to rule out limitations that may limit engagement in walking and standing bouts. An additional criterion for women was that they were not pregnant or planning to become pregnant in the next year.

### Intervention

This was a multicomponent intervention to decrease sedentary behavior in the workplace and comprised organizational, environmental, and individual-level strategies to target behavior change at both the individual and cluster levels [22]. The enrolled participants received initial face-to-face counseling sessions on the benefits of sitting less, standing, and moving more. Supervisors of the participants in the treadmill and sit-to-stand desk groups received initial on-site training on the benefits of decreasing sedentary behavior in the workplace and on strategies to encourage participant engagement in the intervention, which they did at regular department meetings. Participant counseling and supervisor trainings were repeated every 3 months for the treadmill and sit-to-stand desk groups, and these participants were also given feedback on their measured physical behaviors during these sessions.

The height-adjustable desks used in this study were the WorkFit-D from Ergotron Inc. In the treadmill desk group, a WorkFit-D desk was retrofitted with a treadmill (TR1200 DT-3, LifeSpan Fitness Inc) for each participant. Before using the desks, participants were trained to maintain appropriate ergonomic postures while standing and sitting at the height-adjustable desk and were instructed to gradually acclimate to standing and walking while working. These strategies were based on recommendations from the Occupational Safety and Health Administration [39,40] and qualitative feedback from our prior work [41]. Both treadmill and sit-to-stand desk workers were recommended to replace at least 3 hours of daily sedentary time with upright behaviors (ie, 3 hours of standing/day in bouts of 10-30 min after acclimation for sit-to-stand desk participants and 2 hours of walking/day and one hour of standing/day in bouts of 10-30 min after acclimation for treadmill desk participants). The prescribed duration of 10- to 30-minute bouts was recommended to dissuade participants from engaging in prolonged postural fixity, as prolonged static standing has been suggested to result in unfavorable cardiovascular physiology [42,43], which is similar to the detrimental effects observed after prolonged sitting for approximately >30 minutes of continuous sitting [28,36,37]. Seated desk controls were recommended to engage in three 10-minute bouts of moderate to vigorous intensity walking per day to meet the 2008 federal physical activity guidelines [44].

### Data Collection and Measures

#### Overview

Assessments included activity monitoring (activPAL 3C, PAL Technologies Ltd; 7 days on the right thigh during waking hours) at baseline and after 3, 6, and 12 months. In this study, we use the following abbreviations: 3-month follow-up (M3); 6-month follow-up (M6); and 12-month follow-up (M12). The outcome variables described in the subsequent section below are derived from wake-wear data, in which at least 4 valid days of sensor data consisting of a minimum of 10 hours of wake-wear per day from a participant were required for inclusion in the analyses [45]. Detailed procedures for activity monitoring including postprocessing of data have been described previously [22].

#### Outcome Variables

##### Aim 1

Outcome variables for aim 1, which aimed to determine the impact of desk type on resultant patterns of physical behavior, included the number of total daily and workday sedentary, standing, and stepping bouts in durational categories of 1 to 4.9 minutes, 5 to 9.9 minutes, 10 to 19.9 minutes, 20 to 29.9 minutes, 30 to 39.9 minutes, 40 to 49.9 minutes, 50 to 59.9 minutes, and ≥60.0 minutes. Bout durations of 10.0 to 19.9 minutes and 20.0 to 29.9 minutes are henceforth referred to as prescribed bouts. Although sedentary bout lengths have previously been reported to be stratified by bins of various bout lengths (ie, starting >1, >5, >10, >20, >30, or >60 min) [46], this approach does not comprehensively assess the impact of desk type on facilitating sitting less and moving more. Therefore, our bout length stratification scheme aimed to quantify the impact of desk type on the durational patterns of static postural (ie, sitting and standing) and stepping bouts.

##### Aim 2

Outcome variables for aim 2 included usual sedentary, standing, and stepping bout durations for the total day and during the workday. As physical behavior bouts are power law distributed, a nonparametric measure for sedentary bout durations (ie, usual bout durations) has previously been reported as a more representative measure of central tendency [28,38] compared with mean or median sedentary bout durations. We used this nonparametric approach to calculate the usual bout durations for total daily and workday sedentary, standing, and stepping bouts. Details of the procedure for calculating the usual bout durations have been reported previously [38].

### Sample Size

The sample size of 66 participants equally distributed over 3 study arms was based on conservative power estimates of the change in the trial’s primary outcome of total daily sedentary behavior, which we reported previously [22], accounting for a potential loss to follow-up. Details of our power and sample size estimation have been reported previously [22] and are also available in Multimedia Appendix 1.

### Statistical Analyses

All analyses were conducted using SAS 9.4 (SAS Institute Inc). As our outcomes are exploratory, we present a post hoc determination of treatment-response trends for behavior change models indicated by unidirectional 95% CIs, which do not overlap the null value. This exploratory approach avoids confirmatory statistical significance conclusions based on *P* values and thus does not require the application of Bonferroni corrections. Such exploratory analyses are appropriate when the objective is to develop new hypotheses to further study the observed phenomena [47].

We used random-intercept mixed linear models that accounted for repeated measures and clustering effects to assess between-group and within-group differences in the 1-year study period for all outcome variables. Data checks ensured that the underlying assumptions of the statistical modeling used in our data were not violated. Cluster effects for all outcomes were tested by calculating the intraclass correlation coefficients and their statistical significance with α set at <.05. Losses to follow-up were handled as intent-to-treat, and missing data attributable to unsystematic factors (eg, monitoring malfunction, sickness, improper device placement, forgetfulness to wear devices, and log work hours) were imputed using joint multiple imputation [48,49]. Preliminary testing for potential confounding variables (ie, activity monitor wear time and demographics) only detected significant between-group differences for age [22]. Thus, mixed linear models for all outcome variables were adjusted for age. We also tested for statistically significant differences at baseline for all outcome variables using mixed linear models. This testing detected significant (*P*<.05) baseline differences for a select number of variables, including total daily 1- to 4.9-minute sedentary and standing bouts; total daily 50- to 60-minute standing bouts; workday 1- to 4.9-minute, 5- to 9.9-minute, and >60-minute sedentary bouts; workday 1- to 4.9-minute and 50- to 59.9-minute standing bouts; workday 30- to 39.9-minute stepping bouts; and total daily usual sedentary bout duration. Hence, adjustment of analyses for baseline measures was only necessary for these variables during the subsequent mixed linear models on follow-up measures. In this study, we use the symbol “⟟” to denote models that were statistically adjusted for baseline measures. Cohen *d* effect size was calculated for all between- and within-group comparisons and categorized using standardized thresholds (ie, 0.01=very small, 0.2=small, 0.5=medium, 0.8=large, 1.2=very large, and 2.0=huge) [50]. In our results, we use the following abbreviations: very small effect size (VS), small effect size (S), medium effect size (M), large effect size (L), very large effect size (VL), and huge effect size (H).

In addition, sensitivity analyses included participants who completed the 1-year intervention (n total day=42: 13 controls, 13 sit-to-stand desks, and 16 treadmill desks; and n workday=35: 10 controls, 12 sit-to-stand desks, and 13 treadmill desks). These analyses were conducted for all outcomes to determine if the overall treatment-response trends of the 2 interventions were altered when examining an ideal but less conservative scenario of complete intervention compliance and to evaluate the sensitivity of the results to the handling of missing data and covariate adjustment [51].

## Results

### Overview

The control group comprised 20 female and 1 male participants (8 African American/Black, 12 non-Hispanic White, and 1 Hispanic White; mean age 41.9, SD 11.5 years; mean BMI 33.3, SD 5.9 kg/m^2^). The sit-to-stand desk group comprised 21 female and 2 male participants (2 African American/Black, 16 non-Hispanic White, 3 Hispanic White, 1 Asian, and 1 other race or ethnicity; mean age 43.6, SD 12.2 years; mean BMI 30.8, SD 6.0 kg/m^2^). The treadmill desk group comprised 18 female and 4 male participants (5 African Americans/Black, 15 non-Hispanic White, 1 Hispanic White, and 1 other race or ethnicity; mean age 50.4, SD 12.0 years; mean BMI 33.5, SD 4.9 kg/m^2^). Self-reporting of medical history showed a prior or current history of hypertension in 11 participants (8 sit-to-stand desk, 2 treadmill desk, and 1 control), diabetes in 5 participants (3 sit-to-stand desk and 2 treadmill desk), cardiovascular disease in 2 participants (2 treadmill desk), and musculoskeletal conditions in 24 participants (6 sit-to-stand desk, 9 treadmill desk, and 9 controls). Sample sizes after loss to follow-up for the total day and workday analyses are shown in Multimedia Appendices 2 and 3, respectively; the mean activity monitoring times for the total day and workday are shown in Multimedia Appendix 4, and baseline values for all outcome variables are shown in Table 1.

**Table 1:** Baseline outcome variables by group (n=66).

Outcome		Control (n=21)			Sit-to-stand desk (n=23)			Treadmill desk (n=22)	
		Total day, mean (SD)	Workday, mean (SD)	Total day, mean (SD)		Workday, mean (SD)	Total day, mean (SD)		Workday, mean (SD)
**Daily sedentary bouts**									
	Number of daily 1- to 4.9-minute duration sedentary bouts	14.2 (5.9)	12.5 (9.4)	11.3 (5.5)		7.2 (6.5)	9.8 (5.3)		6.5 (7.1)
	Number of daily 5- to 9.9-minute duration sedentary bouts	6.7 (3.2)	5.7 (5.1)	5.5 (2.9)		3.5 (2.9)	5.3 (3)		2.9 (3)
	Number of daily 10- to 19.9-minute duration sedentary bouts	5.8 (2.5)	4 (2.6)	5.8 (2.4)		3.9 (2.2)	5.3 (2.4)		3.1 (2.3)
	Number of daily 20- to 29.9-minute duration sedentary bouts	2.9 (1.3)	1.8 (1.5)	3.1 (1.3)		2 (1.3)	3 (1.3)		2.1 (1.5)
	Number of daily 30- to 39.9-minute duration sedentary bouts	1.6 (0.9)	0.9 (0.9)	1.7 (0.9)		1.2 (0.8)	1.6 (0.9)		1 (0.9)
	Number of daily 40- to 49.9-minute duration sedentary bouts	1.2 (0.7)	0.6 (0.8)	1 (0.6)		0.7 (0.7)	1 (0.7)		0.4 (0.7)
	Number of daily 50- to 59.9-minute duration sedentary bouts	0.6 (0.5)	0.5 (0.7)	0.6 (0.5)		0.5 (0.6)	0.8 (0.5)		0.6 (0.7)
	Number of daily >60-minute duration sedentary bouts	1.3 (1)	0.6 (0.8)	1.4 (0.9)		0.6 (0.7)	1.7 (0.9)		1.2 (1)
**Daily standing bouts**									
	Number of daily 1- to 4.9-minute duration standing bouts	22.6 (9.8)	16.9 (11.1)	16.3 (9.1)		9.6 (8.6)	18.2 (9.1)		9.4 (9.4)
	Number of daily 5- to 9.9-minute duration standing bouts	5.0 (3.9)	4.9 (4.7)	3.3 (3.7)		2.6 (3.6)	5.1 (4.0)		2.9 (3.9)
	Number of daily 10- to 19.9-minute duration standing bouts	1.9 (2.1)	1.8 (2.1)	1.2 (2.0)		0.9 (2.0)	2.0 (2.1)		1.5 (2.1)
	Number of daily 20- to 29.9-minute duration standing bouts	0.4 (0.7)	0.5 (1.0)	0.2 (0.7)		0.3 (0.9)	0.6 (0.7)		0.5 (0.9)
	Number of daily 30- to 39.9-minute duration standing bouts	0.1 (0.3)	0.2 (0.5)	0.2 (0.3)		0.3 (0.5)	0.1 (0.4)		0.2 (0.5)
	Number of daily 40- to 49.9-minute duration standing bouts	0 (0.2)	0.1 (0.2)	0 (0.2)		0 (0.2)	0 (0.2)		0 (0.3)
	Number of daily 50- to 59.9-minute duration standing bouts	0.1 (0.1)	0.1 (0.1)	0 (0.1)		0 (0.1)	0 (0.1)		0 (0.1)
	Number of daily >60-minute duration standing bouts	0.1 (0.3)	0.1 (0.3)	0 (0.3)		0 (0.3)	0.1 (0.3)		0.1 (0.3)
**Daily stepping bouts**									
	Number of daily 1- to 4.9-minute duration stepping bouts	19.6 (7.8)	13 (8)	22.2 (7.6)		11.6 (5.6)	21 (8.1)		9.4 (5.8)
	Number of daily 5- to 9.9-minute duration stepping bouts	5.6 (3)	2 (1.8)	6.7 (3)		2.6 (1.6)	7 (3.1)		2.3 (1.6)
	Number of daily 10- to 19.9-minute duration stepping bouts	2.3 (1.7)	0.7 (1.1)	2.6 (1.7)		1.1 (0.9)	3.2 (1.8)		1 (1)
	Number of daily 20- to 29.9-minute duration stepping bouts	0.5 (0.7)	0.3 (0.5)	0.7 (0.7)		0.2 (0.4)	0.6 (0.8)		0.2 (0.4)
	Number of daily 30- to 39.9-minute duration stepping bouts	0.2 (0.3)	0.2 (0.3)	0.3 (0.3)		0.1 (0.2)	0.2 (0.3)		0 (0.2)
	Number of daily 40- to 49.9-minute duration stepping bouts	0.1 (0.2)	0 (0.1)	0 (0.2)		0 (0.1)	0.1 (0.2)		0 (0.1)
	Number of daily 50- to 59.9-minute duration stepping bouts	0 (0.1)	0 (0.1)	0 (0.1)		0 (0.1)	0 (0.1)		0 (0.1)
	Number of daily >60-minute duration stepping bouts	0.1 (0.2)	0 (0.1)	0.1 (0.1)		0 (0.1)	0 (0.1)		0 (0.1)
**Usual daily bout durations**									
	Usual daily sedentary bout duration (min)	28.7 (12.2)	22 (18.7)	27.9 (11.2)		24.1 (17.1)	34.8 (10.5)		31.3 (19.2)
	Usual daily standing bout duration (min)	4.8 (5.6)	7.4 (7.8)	4.3 (5.5)		4.2 (6.4)	6 (5.7)		5.2 (7)
	Usual daily stepping bout duration (min)	6.9 (4.4)	4.1 (4.3)	7.1 (4.2)		5 (3.9)	9.1 (4.1)		4.7 (4)

### Cluster Effects

The cluster effect did not significantly (all *P* values >.05) account for the variability in any of the outcome variables (Multimedia Appendix 5). Therefore, aim 1 and aim 2 outcome observations for each comparison group (3 randomization groups × 4 time points) were analyzed at the participant level instead of cluster, and the final statistical analyses accounted for the random effect of cluster in the study design.

### Aim 1

For aim 1, we present key findings, followed by detailed findings for the frequency of total day and workday sedentary, standing, and stepping bouts categorized by bout durations.

#### Aim 1 Key Findings

Key findings of our stratified behavioral bout durations analyses included the following:

1. Sedentary bouts: the treadmill desk group engaged in fewer short-duration bouts of <20 minutes relative to the control and sit-to-stand desk groups after M12, but the sit-to-stand desk group had fewer >60-minute bouts relative to the control and treadmill desk groups after M3, M6, and M12.
2. Standing bouts: the treadmill desk group engaged in more prescribed duration (10-30 min) bouts, relative to controls after M6 and M12, and also more prolonged (>30 min) bouts after M3 and M12, relative to controls and the sit-to-stand desk group. In contrast, the sit-to-stand desk group favored increasing standing in prescribed bouts or shorter (1-10 min) after M3 and M12.
3. Stepping bouts: the treadmill desk group engaged in more bouts of prescribed (10-30 min) or shorter durations (1-10 min) as well as longer durations (>30 min), relative to controls and the sit-to-stand desk group at M3, which was not sustained through M12.

#### Sedentary Bouts

##### Total Day

The treadmill desk group demonstrated trends in accumulating fewer sedentary bouts in categorizations that were <20 minutes, relative to both the control (mean Δ in 1- to 4.9-min duration bouts at M12: −5.0 bouts/day, 95% CI −9.0 to −0.9; *P*=.02; L) and sit-to-stand desk groups (mean Δ in 1- to 4.9-min duration bouts at M12: −6.8 bouts/day, 95% CI −10.8 to −2.9; *P*=.001; L and mean Δ in 10- to 19.9-min duration bouts at M12: −2.3 bouts/day, 95% CI −3.9 to −0.8; *P*=.004; L; Multimedia Appendix 6). Conversely, the sit-to-stand desk group demonstrated trends in accumulating fewer prolonged sedentary bouts that were >60 minutes, relative to both the control (mean Δ at M6: −1.1 bouts/day, 95% CI −1.9 to −0.3; *P*=.008; L and mean Δ at M12: −0.7 bouts/day, 95% CI −1.3 to −0.1; *P*=.02; M) and treadmill desk groups (mean Δ at M12: −0.8 bouts/day, 95% CI −1.4 to −0.2; *P*=.008; L; Multimedia Appendix 6).

Within-group trends for favorable treatment responses were observed, where both the treadmill and sit-to-stand desk groups demonstrated shifts toward engaging in shorter sedentary bouts through an increase in the number of sedentary bouts of 1 to 4.9 minutes. Although these short-duration bouts increased in the treadmill desk group after M6, the favorable trend disappeared at M12. Conversely, the sit-to-stand desk group successfully maintained this favorable trend after M12 (Multimedia Appendix 6). In addition, the treadmill desk group showed an unfavorable within-group trend of increasing prolonged sedentary bouts of 40 to 49.9 minutes after M6 but reversed this trend after M12 (Multimedia Appendix 6).

##### Workday

Similar to the total daily bouts, the treadmill desk group demonstrated trends of accumulating fewer workday sedentary bouts in categorizations that were <20 minutes, relative to both the control (mean Δ in 1- to 4.9-min duration bouts at M12: −6.9 bouts/day, 95% CI −11.7 to −2.1; *P*=.005; L and mean Δ in 10- to 19.9-min duration bouts at M12: −1.8 bouts/day, 95% CI −3.3 to −0.3; *P*=.02; M) and sit-to-stand desk groups (mean Δ in 1- to 4.9-min duration bouts at M12: −5.1 bouts/day, 95% CI −8.8 to −1.3; *P*=.008; L; mean Δ in 5- to 9.9-min duration bouts at M3: −1.9 bouts/day, 95% CI −3.6 to −0.2; *P*=.03; M; mean Δ in 5- to 9.9-min duration bouts at M12: −2.1 bouts/day, 95% CI −3.9 to −0.4; *P*=.02; M; and mean Δ in 10- to 19.9-min duration bouts at M12: −2.1 bouts/day, 95% CI −3.5 to −0.7; *P*=.004; L; Multimedia Appendix 6). In addition, the treadmill desk group demonstrated short-term trends of accumulating a higher number of workday prolonged sedentary bouts of 50 to 59.9 minutes, relative to both the control (mean Δ at M3: 0.9 bouts/day, 95% CI 0.1-1.6; *P*=.02; M) and sit-to-stand desk groups (mean Δ at M3: 0.9 bouts/day, 95% CI 0.2-1.6; *P*=.01; M; Multimedia Appendix 6). In contrast, the sit-to-stand desk group demonstrated a short-term trend of accumulating fewer workday prolonged sedentary bouts of >60 minutes, relative to controls (mean Δ at M3: −0.7 bouts/day, 95% CI −1.2 to−0.1; *P*=.02; L; Multimedia Appendix 6). However, these short-term trends were not sustained beyond M3 in both treatment groups (Multimedia Appendix 6).

Within-group workday trends showed an unfavorable short-term response for the treadmill desk group, with daily increases of prolonged 40 to 49.9 minutes of sedentary bouts after M3, but this was not sustained after M6 or M12 (Multimedia Appendix 6). The treadmill desk group also demonstrated within-group trends of decreasing the number of workday 1- to 4.9-minute and 20- to 29.9-minute sedentary bouts after M12 (Multimedia Appendix 6).

#### Standing Bouts

##### Total Day

The treadmill desk group demonstrated trends of accumulating more prescribed standing bouts in the range of 20 to 29.9 minutes relative to the controls at M6 (mean Δ: 0.6 bouts/day, 95% CI 0.1-1.1; *P*=.03; M) and accumulating fewer daily standing bouts in the range of 1 to 4.9 minutes relative to the sit-to-stand desk group at M12 (mean Δ: −6.9 bouts/day, 95% CI −12.9 to −0.9; *P*=.02; M; Multimedia Appendix 7). In addition, the treadmill desk group showed trends favoring prolonged standing (ie, >30 min) after M3 and sustained this trend through M12 relative to both the control and sit-to-stand desk groups (Multimedia Appendix 7). Specifically, for bouts between 30 and 59.9 minutes, trends sustained in the treadmill desk group after M12 relative to the control and sit-to-stand desk groups were as follows:

1. Mean Δ in 30 to 39.9 minute duration bouts: relative to the controls: 0.4 (95% CI 0.1-0.6; *P*=.003; L); relative to the sit-to-stand desk group: 0.3 (95% CI 0.1-0.5; *P*=.02; M; Multimedia Appendix 7).
2. Mean Δ in 40 to 49.9 minute duration bouts: 0.2 (95% CI 0.1-0.4; *P*=.001; L) bouts relative to both groups (Multimedia Appendix 7)
3. Mean Δ in 50 to 59.9 minute duration bouts: 0.1 (95% CI 0.1-0.2; *P*=.001; L) bouts relative to the sit-to-stand desk group (Multimedia Appendix 7).

For standing bouts of >60 minutes, the treadmill desk group demonstrated trends favoring more accumulation of such bouts relative to both the control (mean Δ: 0.5 bouts/day, 95% CI 0.3-0.7; *P*<.001; VL) and sit-to-stand desk groups at M3 (mean Δ: 0.6 bouts/day, 95% CI 0.3-0.8; *P*<.001; VL), but sustained this trend only relative to controls after M12 (mean Δ: 0.2 bouts/day, 95% CI 0.03-0.4; *P*=.02; M; Multimedia Appendix 7).

Correspondingly, within-group trends showed that the sit-to-stand desk group increased the number of prescribed standing bouts at M3 (ie, 10- to 29.9-min bouts; Multimedia Appendix 7). Increases in shorter standing bouts of 1 to 9.9 minutes were also observed for the sit-to-stand desk group at M12 (Multimedia Appendix 7). In contrast, the treadmill desk group favored increases in prolonged bouts at both M3 (ie, 30- to 49.9-min bouts) and M12 (ie, 50- to 59.9-min bouts; Multimedia Appendix 7).

##### Workday

The treadmill desk group demonstrated a trend of accumulating more workday prescribed standing bouts after M6, relative to the sit-to-stand desk group (mean Δ in 20- to 29.9-min duration bouts: 1.0 bouts/day, 95% CI 0.3-1.8; *P*=.01; L), and sustained this trend after M12 (mean Δ: 0.9 bouts/day, 95% CI 0.2-1.5; *P*=.009; L; Multimedia Appendix 7). The trend of the treadmill desk group favoring prolonged standing bouts, which was observed over the total day, was also present for the workday (Multimedia Appendix 7). Specifically, the treadmill desk group demonstrated trends of accumulating more workday standing bouts of the following:

1. Bouts of 30 to 39.9 minutes duration, relative to the sit-to-stand desk group at M6 (mean Δ: 0.6 bouts/day, 95% CI 0.2-1.0; *P*=.007; L) and M12 (mean Δ: 0.4 bouts/day, 95% CI 0.1-0.7; *P*=.02; M), and controls at M12 (mean Δ: 0.5 bouts/day, 95% CI 0.1-0.8; *P*=.006; L).
2. Bouts of 40 to 49.9 minute duration, relative to the sit-to-stand desk group at M12 (mean Δ: 0.3 bouts/day, 95% CI 0.1-0.5; *P*=.002; L).
3. Bouts of 50 to 59.9 minute duration, relative to both controls (mean Δ: 0.2 bouts/day, 95% CI 0.1-0.3; *P*<.001; VL) and the sit-to-stand desk group at M12 (mean Δ: 0.2 bouts/day, 95% CI 0.1-0.3; *P*=.001; VL).
4. Bout >60 minute in duration, relative to both controls (mean Δ: 0.6 bouts/day, 95% CI 0.2-0.9; *P*=.002; L) and the sit-to-stand desk group at M3 (mean Δ: 0.7 bouts/day, 95% CI 0.3-1.0; *P*<.001; L), which was sustained relative to controls after M12 (mean Δ: 0.3 bouts/day, 95% CI 0.1-0.5; *P*=.007; L).

Correspondingly, within-group workday trends showed the treadmill desk group increasing both prescribed and prolonged standing bouts (ie, 10-19.9 min and 20-20.9 min at M12, 30-39.9 min at both M6 and M12, 40-49.9 min at M12, 50-59.9 min at M12, and >60 min at M3; Multimedia Appendix 7). In contrast, the sit-to-stand desk group favored workday increases in both short (ie, 1-4.9 min and 5-9.9 min at M3) and prescribed standing bouts (ie, 10-19.9 min at both M3 and M12; Multimedia Appendix 7).

#### Stepping Bouts

##### Total Day

Similar to the standing bout trends, the treadmill desk group demonstrated a short-term (M3) trend of accumulating longer-duration (ie, 40-49.9 min) stepping bouts relative to both the control (mean Δ: 0.1 bouts/day, 95% CI 0.0002-0.2; *P*=.05; M) and sit-to-stand desk groups (mean Δ: 0.2 bouts/day, 95% CI 0.04-0.2; *P*=.007; L), but these trends were not sustained through M6 or M12 (Multimedia Appendix 8). Conversely, the sit-to-stand desk group demonstrated a trend of accumulating short-duration (ie, 1-4.9 min) stepping bouts relative to controls after M6 (mean Δ: 5.6 bouts/day, 95% CI 0.3-10.9; *P*=.04; M), but this trend was not sustained after M12 (Multimedia Appendix 8).

Within-group trends suggest potential for gains in both treatment groups because of increasing trends in the number of stepping bouts accumulated across multiple stepping bouts durations (ie, 1-4.9, 10-19.9, 20-29.9, 30-39.9, 40-49.9, 50-59.9, and >60 min) after M6 (Multimedia Appendix 8). However, only the sit-to-stand desk group sustained such trends after M12, and these increases were only observed for shorter-duration stepping categories (ie, <20 min; Multimedia Appendix 8). The control group also demonstrated within-group trends of increasing the number of stepping bouts lasting 5 to 9.9, 10 to 19.9, and 30 to 39.9 minutes after M6, but these were not sustained after M12 (Multimedia Appendix 8).

##### Workday

The treadmill desk group demonstrated short-term workday trends of accumulating more stepping in bouts that were equal to or shorter than the 30 minutes relative to both controls (ie, mean Δ in 5-9.9 min duration bouts at M3: 1.5 bouts/day, 95% CI 0.3-2.7; *P*=.02; M and mean Δ in 10-19.9 min duration bouts at M3: 0.8 bouts/day, 95% CI 0.1-1.6; *P*=.03; M) and the sit-to-stand desk group (mean Δ in 20-29.9 min duration bouts at M3: 0.4 bouts/day, 95% CI 0.1-0.7; *P*=.01; M and mean Δ in 20-29.9 min duration bouts at M6: 0.4 bouts/day, 95% CI 0.1-0.7; *P*=.01; L; Multimedia Appendix 8). The treadmill desk group also demonstrated short-term workday trends of increasing the accumulation of long duration stepping bouts relative to the sit-to-stand desk group (mean Δ in 30-39.9 min duration bouts at M3: 0.2 bouts/day, 95% CI 0.1-0.4; *P*=.005; L and mean Δ in 40-49.9 min duration bouts at M3: 0.2 bouts/day, 95% CI 0.1-0.4; *P*=.001; L; Multimedia Appendix 8). In addition, the control group demonstrated a M12 workday trend of accumulating more 50 to 59.9 minutes stepping bouts relative to the sit-to-stand desk group (mean Δ: 0.2 bouts/day, 95% CI 0.1-0.3; *P*<.001; VL; Multimedia Appendix 8).

Correspondingly, within-group workday trends showed that the treadmill desk group had short-term increases in 20 to 29.9 minutes stepping bouts after both M3 and M6, which were not sustained after M12, as well as M3 and M12 increases in multiple longer-duration stepping bout categorizations (ie, 30-39.9 min at M3 and M12, 40-49.9 min at M3, 50-59.9 min at M12, and >60 min at M6; Multimedia Appendix 8). In addition, the control group showed an anomalous within-group increase in 50 to 59.9 min stepping bouts at M12 (Multimedia Appendix 8).

### Aim 2

For aim 2, we present the key findings, followed by detailed findings for total day and workday usual sedentary, standing, and stepping bout durations.

#### Aim 2 Key Findings

Key findings of our usual bout duration analyses included the following:

1. Sedentary bouts: the treadmill desk group had longer usual bout durations after M12 compared with controls and the sit-to-stand desk group, which had shorter usual bout durations compared with controls at M3, which were not sustained through M12.
2. Standing bouts: both treatment groups had longer usual bout durations compared with controls after M3 and M12, and the treadmill desk group had longer usual bout durations compared with the sit-to-stand desk group after M3, which was not sustained through M12.
3. Stepping bouts: the treadmill desk group had longer usual bout durations relative to controls and the sit-to-stand desk group after M3, but this trend was only sustained after M12 relative to the sit-to-stand desk group.

#### Usual Sedentary Bout

##### Total Day

Relative to controls, the sit-to-stand desk group demonstrated a trend of shorter usual sedentary bout durations after M3 (mean Δ: −10.1 min/bout, 95% CI −17.9 to−2.2; *P*=.01; L), which was not sustained after M6 or M12 (Table 2). In contrast, the treadmill desk group demonstrated a trend of longer usual sedentary bout durations after M12 relative to both the control (mean Δ: 9.0 min/bout, 95% CI 1.6-16.4; *P*=.02; M) and sit-to-stand desk groups (mean Δ: 10.9 min/bout, 95% CI 3.5-18.3; *P*=.004; L; Table 2). Nevertheless, reductions in usual sedentary bout duration within the treadmill desk group after M3 (mean Δ: −6.8 min/bout, 95% CI −13.3 to −0.2; *P*=.04; M) suggests the potential for a favorable short-term treatment response (Table 2).

**Table 2:** Between- and within-group comparisons of usual daily physical behavior bout durations over the total day and workday, adjusted for age.^a^

Outcome				Control				Sit-to-stand desk			Treadmill desk					Sit-to-stand desk—control					Treadmill desk—control					Treadmill—sit-to-stand desk			
				Change from baseline (95% CI)		Effect size (*P* value)		Change from baseline (95% CI)		Effect size (*P* value)	Change from baseline (95% CI)			Effect size (*P* value)	Difference (95% CI)			Effect size (*P* value)		Difference (95% CI)			Effect size (*P* value)		Difference (95% CI)			Effect size (*P* value)	
**Usual daily sedentary bout duration (min)**																													
	**M3^b^**																												
		Total day	6.5 (−1.3 to 14.3)		0.52^c^ (.10)		−2.8 (−8.9 to 3.3)		0.25^d^ (.36)			−6.8 (−13.3 to −0.2)	0.56^c^ (.04^e^)				−10.1 (−17.9 to −2.2)		0.85^f^ (.01^e,g^)			−8.5 (−16.9 to 0)		0.65^c^ (.05^g^)			1.6 (−5.8 to 9.0)		0.13^h^ (.67^g^)
		Workday	20 (2.9 to 37)		0.78^c^ (.02^e^)		−2.5 (−11.8 to 6.8)		0.14^h^ (.60)			−2.3 (−14.1 to 9.6)	0.10^h^ (.71)				−20.3 (−37.7 to −2.9)		0.81^f^ (.02^e^)			−12.9 (−31.5 to 5.7)		0.46^d^ (.17)			7.4 (−5.2 to 20.1)		0.35^d^ (.25)
	**M6^i^**																												
		Total day	1.7 (−9.2 to 12.5)		0.10^h^ (.76)		−3.2 (−9.7 to 3.2)		0.28^d^ (.36)			−2.8 (−10.1 to 4.6)	0.22^d^ (.46)				−5.6 (−16.9 to 5.6)		0.36^d^ (.33^g^)			0.4 (−11.5 to 12.2)		0.02^h^ (.95^g^)			6.0 (−2.3 to 14.4)		0.46^d^ (.16^g^)
		Workday	−1.1 (−17.9 to 15.8)		0.04^h^ (.90)		−7.4 (−16.8 to 2.1)		0.43^d^ (.13)			−1.0 (−13.7 to 11.7)	0.05^h^ (.88)				−4.2 (−21.4 to 13.1)		0.17^h^ (.64)			9.4 (−9.7 to 28.5)		0.34^d^ (.33)			13.5 (0.1 to 27.0)		0.63^c^ (.049^e^)
	**M12^j^**																												
		Total day	−0.6 (−7.4 to 6.2)		0.05^h^ (.87)		−2.0 (−8.4 to 4.4)		0.17^h^ (.54)			3.4 (−2.9 to 9.7)	0.30^d^ (.29)				−1.9 (−9.1 to 5.3)		0.17^h^ (.61^g^)			9 (1.6 to 16.4)		0.79^c^ (.02^e,g^)			10.9 (3.5 to 18.3)		0.92^f^ (.004^e,g^)
		Workday	7.0 (−5.3 to 19.4)		0.34^d^ (.26)		−1.3 (−11.0 to 8.3)		0.08^h^ (.79)			0.4 (−10.9 to 11.7)	0.02^h^ (.95)				−6.2 (−19.1 to 6.7)		0.31^d^ (.34)			2.7 (−11.5 to 16.9)		0.12^h^ (.71)			8.9 (−3.4 to 21.2)		0.45^d^ (.16)
**Usual daily standing bout duration (min)**																													
	**M3**																												
		Total day	0.8 (−2.3 to 3.9)		0.14^h^ (.62)		1.3 (−1.4 to 4.0)		0.24^d^ (.34)			6.6 (3.0 to 10.1)	0.92^f^ (<.001^e^)				0.0 (−3.7 to 3.6)		0.01^h^ (.98)			6.9 (2.5 to 11.4)		0.98^f^ (.002^e^)			7.0 (2.8 to 11.2)		0.98^f^ (.001^e^)
		Workday	−1.6 (−5.5 to 2.4)		0.22^d^ (.44)		1.9 (−1.2 to 5.0)		0.29^d^ (.23)			9.6 (3.6 to 15.5)	0.85^f^ (.002^e^)				0.2 (−4.1 to 4.5)		0.03^h^ (.93)			8.9 (2.1 to 15.7)		0.80^f^ (.01^e^)			8.7 (2.1 to 15.3)		0.79^c^ (.009^e^)
	**M6**																												
		Total day	−0.6 (−4.0 to 2.7)		0.11^h^ (.71)		−0.4 (−3.2 to 2.4)		0.08^h^ (.77)			1.2 (−2.1 to 4.4)	0.18^h^ (.49)				−0.4 (−4.3 to 3.6)		0.06^h^ (.86)			2.9 (−1.5 to 7.3)		0.45^d^ (.19)			3.3 (−0.7 to 7.3)		0.52^c^ (.11)
		Workday	−1.6 (−6.7 to 3.4)		0.20^d^ (.53)		0.3 (−2.9 to 3.5)		0.04^h^ (.88)			3.7 (−0.5 to 7.9)	0.46^h^ (.08)				−1.3 (−6.7 to 4.0)		0.17^h^ (.62)			3.2 (−2.9 to 9.2)		0.36^d^ (.31)			4.5 (−0.5 to 9.5)		0.57^c^ (.08)
	**M12**																												
		Total day	−0.4 (−3.3 to 2.4)		0.08^h^ (.77)		4.3 (0.8 to 7.9)		0.66^c^ (.02^e^)			3.0 (−0.1 to 6.0)	0.49^d^ (.06)				4.2 (0.1 to 8.3)		0.65^c^ (.046^e^)			4.5 (0.7 to 8.4)		0.76^c^ (.02^e^)			0.4 (−4.0 to 4.7)		0.05^h^ (.87)
		Workday	−1.8 (−5.7 to 2.1)		0.25^d^ (.36)		2.5 (−0.8 to 5.8)		0.38^d^ (.14)			6.2 (2.3 to 10.1)	0.80 ^f^ (.002^e^)				1.1 (−3.3 to 5.4)		0.16^h^ (.63)			5.8 (0.9 to 10.6)		0.76^c^ (.02^e^)			4.7 (−0.1 to 9.5)		0.61^c^ (.05)
**Usual daily stepping bout duration (min)**																													
	**M3**																												
		Total day	−0.3 (−3.0 to 2.4)		0.06^h^ (.85)		0.2 (−2.2 to 2.6)		0.05^h^ (.87)			−1.2 (−3.6 to 1.2)	0.29^d^ (.31)				0.6 (−2.2 to 3.4)		0.15^h^ (.66)			1.2 (−1.6 to 4.1)		0.28^d^ (.40)			0.6 (−2.0 to 3.3)		0.14^h^ (.65)
		Workday	0.4 (−2.3 to 3.0)		0.09^h^ (.77)		−0.3 (−2.4 to 1.7)		0.09^h^ (.74)			4.6 (1.8 to 7.5)	0.88 ^f^ (.002^e^)				0.1 (−2.7 to 2.9)		0.03^h^ (.94)			4.8 (1.3 to 8.3)		0.88 ^f^ (.007^e^)			4.7 (1.6 to 7.8)		0.89 ^f^ (.003^e^)
	**M6**																												
		Total day	1 (−2.2 to 4.2)		0.21^d^ (.53)		0.8 (−1.7 to 3.3)		0.19^h^ (.52)			−1.7 (−4.6 to 1.2)	0.34^d^ (.31)				0.0 (−3.4 to 3.3)		0.01^h^ (.98)			−0.5 (−4.2 to 3.2)		0.09^h^ (.79)			−0.5 (−3.6 to 2.7)		0.09^h^ (.78)
		Workday	3 (−2.5 to 8.5)		0.39^d^ (.28)		0.0 (−2.1 to 2.2)		0.01^h^ (.97)			5.6 (0.9 to 10.3)	0.72^c^ (.02^e^)				−2.1 (−7.8 to 3.5)		0.28^d^ (.46)			3.2 (−3.9 to 10.2)		0.31^d^ (.38)			5.3 (0.3 to 10.3)		0.68^c^ (.04^e^)
	**M12**																												
		Total day	1.1 (−1.5 to 3.7)		0.24^d^ (.42)		−0.1 (−2.5 to 2.2)		0.03^h^ (.92)			−2.1 (−4.5 to 0.3)	0.50^c^ (.09)				−1.0 (−3.7 to 1.6)		0.25^d^ (.45)			−1.0 (−3.7 to 1.8)		0.22^d^ (.50)			0.1 (−2.6 to 2.7)		0.02^h^ (.96)
		Workday	1.6 (−0.9 to 4.2)		0.37^d^ (.21)		−0.5 (−2.6 to 1.5)		0.14^h^ (.61)			2.7 (0.2 to 5.3)	0.59^c^ (.04^e^)				−1.3 (−4.0 to 1.4)		0.31^d^ (.34)			1.7 (−1.5 to 4.8)		0.34^d^ (.29)			3 (0.1 to 5.9)		0.65^c^ (.04^e^)

^a^Sample sizes after losses to follow-up: baseline n=66 (21 controls, 23 sit-to-stand desks, and 22 treadmill desks), M3 n=58 (15 controls, 21 sit-to-stand desks, and 22 treadmill desks), M6 n=53 (14 controls, 20 sit-to-stand desks, and 19 treadmill desks), and M12 n=58 (18 controls, 20 sit-to-stand desks, and 20 treadmill desks).

^b^M3: 3-month follow-up.

^c^Medium effect size.

^d^Small effect size.

^e^Effect trend (ie, unidirectional 95% CIs not overlapping null value).

^f^Large effect size.

^g^Adjusted for baseline.

^h^Very small effect size.

^i^M6: 6-month follow-up.

^j^M12: 12-month follow-up.

##### Workday

The difference in the sit-to-stand desk group’s workday usual sedentary bout duration, relative to controls, after M3 (mean Δ: −20.3 min/bout, 95% CI −37.7 to −2.9; *P*=.02; L) was greater than that observed over the total day. However, this trend was not sustained after M6 or M12 (Table 2). In addition, the treadmill desk group demonstrated a trend of longer workday usual sedentary bout durations relative to the sit-to-stand desk group after M6 (mean Δ: 13.5 min/bout, 95% CI 0.1-27.0; *P*=.049; M; Table 2). Interestingly, a major contributing factor to the between-group workday trend observed between the sit-to-stand desk and control groups after M3 was owing to a 20-minute increase (95% CI 2.9-37; *P*=.02; M) in the usual sedentary bout durations in the control group, but this trend returned to baseline after M6 before increasing again after M12 by about half as much time on average per bout relative to the short-term increase (Table 2).

#### Usual Standing Bout

##### Total Day

Both treatment groups demonstrated a trend of longer usual standing bout durations than the control group (Table 2). For the treadmill desk group, this trend was observed after M3 (mean Δ from controls: 6.9 min/bout, 95% CI 2.5-11.4; *P*=.002; L), which was sustained at M12 (mean Δ from controls: 4.5 min/bout, 95% CI 0.7-8.4; *P*=.02; M). Relative to the control group, the sit-to-stand desk group demonstrated this trend at M12 (mean Δ: 4.2 min/bout, 95% CI 0.1-8.3, *P*=.046; M; Table 2). The treadmill desk group also demonstrated a trend of longer usual standing bouts relative to the sit-to-stand desk group after M3 (mean Δ: 7.0 min/bout, 95% CI 2.8-11.2; *P*=.001; L), but this was not sustained after M6 or M12 (Table 2). The greatest increase in the usual standing bout durations within the treadmill desk group occurred after M3 (mean Δ: 6.6 min/bout, 95% CI 3.0-10.1; *P*<.001; L), whereas in the sit-to-stand desk group, it occurred after M12 (mean Δ: 4.3 min/bout, 95% CI 0.8-7.9; *P*=.02; M; Table 2).

##### Workday

The treadmill desk group demonstrated trends of longer workday usual standing bout durations after M3 relative to both the control (mean Δ: 8.9 min/bout, 95% CI 2.1-15.7; *P*=.01; L) and sit-to-stand desk groups (mean Δ: 8.7 min/bout, 95% CI 2.1-15.3; *P*=.009; M); however, these trends were sustained only relative to the controls at M12 (mean Δ: 5.8 min/bout, 95% CI 0.9-10.6; *P*=.02; M; Table 2). The treadmill desk group had its greatest within-group increase in workday usual standing bout duration after M3 (mean Δ: 9.6 min/bout 95% CI 3.6-15.5; *P*=.002; L) and sustained this after M12 (mean Δ: 6.2 min/bout, 95% CI 2.3-10.1; *P*=.002; L; Table 2). No such trends were observed in the sit-to-stand desk group (Table 2).

#### Usual Stepping Bout

##### Total Day

No between-group or within-group trends for the usual stepping bout duration were observed for either group.

##### Workday

After M3, the treadmill desk group demonstrated trends of longer workday usual stepping bouts relative to both the control (mean Δ: 4.8 min/bout, 95% CI 1.3-8.3; *P*=.007; L) and sit-to-stand desk groups (mean Δ: 4.7 min/bout, 95% CI 1.6-7.8; *P*=.003; L; Table 2). These trends were sustained only relative to the sit-to-stand desk group after M6 (mean Δ: 5.3 min/bout, 95% CI 0.3-10.3; *P*=.04; M) and M12 (mean Δ: 3.0 min/bout, 95% CI 0.1-5.9; *P*=.04; M; Table 2). Correspondingly, the treadmill desk group increased its workday usual stepping bout duration relative to baseline after each follow-up, the largest of which occurred after M6 (mean Δ: 5.6 min/bout, 95% CI 0.9-10.3; *P*=.02; M; Table 2). Such within-group trends in the usual workday stepping bout duration did not occur in the sit-to-stand desk group (Table 2).

### Sensitivity Analyses

The baseline characteristics for aim 1 and 2 outcomes of the completers’ analyses are shown in Multimedia Appendix 9.

#### Sedentary Behavior Bout Outcomes

Compared with the intent-to-treat analyses, the sedentary bout pattern trends that prevailed in the completers’ analyses were as follows:

1. The treadmill desk group accumulated fewer daily sedentary bouts of <20 minutes, relative to both controls and the sit-to-stand desk group after M12 during both the total day and workday (Multimedia Appendix 10).
2. The treadmill desk group accumulated more prolonged sedentary bouts of >60 minutes, relative to both controls and the sit-to-stand desk groups after M12 over the total day (Multimedia Appendix 10).
3. The sit-to-stand desk group increased short-duration (ie, 1-4.9 min) sedentary bouts, whereas the treadmill desk group decreased such bouts over the total day. The treadmill desk group also decreased 20- to 29.9-minute bouts over the workday from baseline to M12 (Multimedia Appendix 10).
4. The treadmill desk group demonstrated trends of longer usual sedentary bouts, relative to the sit-to-stand desk group after M12 over the total day (Multimedia Appendix 10).

No other sedentary behavior pattern trends were observed for the complete-case analyses.

#### Standing Behavior Bout Outcomes

Compared with the intent-to-treat analyses, the standing bout pattern trends that prevailed in the completers’ analyses were as follows:

1. The treadmill desk group demonstrated daily increases in both the number of prescribed (workday) and prolonged (total day) duration standing bout from baseline to M12 (Multimedia Appendix 10).
2. The treadmill desk group increased its usual standing bout duration from baseline to M12 both over the total day and workday (Multimedia Appendix 10).

No other standing behavior pattern trends were observed for the complete-case analyses.

#### Stepping Behavior Bouts Outcomes

Compared with the intent-to-treat analyses, only a few stepping bout pattern trends prevailed in the completer’s analyses and included the following:

1. The sit-to-stand desk group demonstrated increases in shorter-duration stepping bout categories (ie, <20 min) from baseline to M12 over the total day (Multimedia Appendix 10).
2. The treadmill desk group increased its usual stepping bout duration from baseline to M12 both over the total day and workday (Multimedia Appendix 10).

No other stepping behavior pattern trends were observed for the complete-case analyses.

## Discussion

### Overview

Previously, we reported that the use of both sit-to-stand and treadmill workstations resulted in positive shifts from sedentary behavior in favor of increased daily standing and stepping, such that treadmill desk users engaged in fewer total daily and workday sedentary bouts and sit-to-stand desk users transitioned to upright physical behaviors more frequently both over the total day and workday [22]. Expanding on our previous work, this study suggests variability in the pattern of accumulating sitting, standing, and stepping when using treadmill and sit-to-stand desks.

### Principal Findings and Comparisons With Prior Work

#### Sedentary Bout Patterns

Categorized sedentary bout analyses revealed two key findings: (1) the treadmill desk resulted in fewer daily short-duration (ie, <20 min) sedentary bouts compared with the control and sit-to-stand desk groups after M12 (ie, approximately 4-5 less bouts/total day and approximately 2-4 less bouts/workday), and (2) the sit-to-stand desk resulted in fewer sedentary bouts of >60 minutes compared with the control and treadmill desk groups after M3 (ie, approximately 0.7 less bouts/workday) and after M6 and M12 (ie, approximately 0.7-1 less bouts/total day). Correspondingly, these patterns impacted usual sedentary bout durations such that (1) the treadmill desk group’s total daily usual sedentary bout duration was approximately 9 to 11 minutes longer compared with the control and sit-to-stand desk groups after M12, and (2) the sit-to-stand desk group’s total daily usual sedentary bout duration was approximately 10 minutes shorter than the control groups at M3, which, however, was not sustained through M12. Similar trends for workday usual sedentary bout durations were evident for both intervention groups. However, although the observed workday difference for usual sedentary bout was larger as compared with the total day, these trends were observed only after M3 for the sit-to-stand desk group (ie, approximately 20 min shorter than the control group) and after M6 for the treadmill desk group (ie, approximately 13.5 min longer than sit-to-stand desk group).

The patterns of sedentary behavior bout accumulation observed in the sit-to-stand desk group were consistent with those reported in previous studies [27,28] (ie, sit-to-stand desk reduced prolonged sedentary bouts in relation to controls), but our study resulted in larger differences in usual sedentary bout duration relative to controls. Published findings from 2 cluster randomized sit-to-stand desk trials with a follow-up of at least 12 months reported significant short- and long-term differences in workday time spent in prolonged bouts of sedentary behavior, which was defined as >30 minutes, favoring the intervention groups (ie, 73 less min/workday after 3 months [28], and 35 and 45 less min/workday after 6 and 12 months, respectively, in such bouts [27]). This is consistent with our finding that sit-to-stand desks reduced the accumulation of prolonged sedentary bouts. One of these studies also reported significant short-term differences in the duration of workday usual sedentary bouts in sit-to-stand desk users relative to controls (ie, −4.4 min) [28], which is consistent with but shorter than the duration found in our study, which was −20.3 minutes.

This study is the first to report the impact of treadmill desks on both the distribution of sedentary bouts across temporal categories and usual sedentary bout duration, and the first to provide a head-to-head comparison of such metrics with sit-to-stand desks. An important finding is that treadmill desks exerted a variable response as compared with sit-to-stand desks by favoring the accumulation of sedentary behavior in prolonged bouts when the desk is not being used for walking or standing. This finding is noteworthy because prolonged sedentary behavior may be adversely associated with chronic disease risk factors and overall health [52]. Future research on these 2 types of interventions also needs to examine whether reducing short bouts of sedentary behavior while keeping total volume constant exerts a varying effect on health as compared with reducing prolonged bouts of sedentary behavior.

#### Standing Bout Patterns

Although the treadmill desk group engaged in more prescribed bouts (ie, 10-30 min) relative to controls after M6 and M12 over both the total day and workday (approximately 0.6-1.0 bout/day), an unintended response of engaging in more prolonged duration (ie, >30 min) standing bouts was observed among treadmill desk users. Specifically, treadmill desk users demonstrated trends of engaging in approximately 0.6 to 0.9 more prolonged standing bouts per day, relative to controls and sit-to-stand desk groups over both the total day and workday after M3 and sustained after M12. In contrast, sit-to-stand desk users were more compliant with the recommended intervention by increasing standing in prescribed bouts or shorter (ie, 1-10 min) after M3 and sustained this after M12 over both the total day (ie, approximately 1-2 bouts/day) and workday (approximately 3-8 bouts/day). These findings are noteworthy because prolonged static standing may be detrimental to cardiovascular health [42,43]. We are unsure why users of treadmill workstations preferred to engage in a much higher number of prolonged standing bouts as compared with the sit-to-stand desk group. Poststudy interviews were unable to discern the cause of this behavior. Although prolonged standing was not an issue in our sit-to-stand desk group, future active workstation interventions should consider combining a higher frequency of reinforcing messages to dissuade prolonged standing bouts, in addition to avoiding prolonged sitting among users of both desk types.

Total day standing bout pattern distributions impacted total daily usual standing bout durations such that (1) both treatment groups demonstrated longer total daily usual standing bouts relative to controls after M3 and M12, where the increase ranged from approximately 4 to 7 minutes per bout, and (2) the treadmill desk group’s total daily usual standing bout duration was approximately 7 minutes longer than that of the sit-to-stand desk group at M3, but this trend was not sustained through M12. Similar increasing workday trends were evident when comparing the treadmill desk group with controls and sit-to-stand desk users after M3, where the increase ranged from approximately 6 to 9 minutes per bout, but these trends were only sustained through M12 in relation to the control group.

#### Stepping Bout Patterns

Although the impact on stepping bout patterns was also variable between active workstation types, both sit-to-stand and treadmill desks had less impact on the overall patterns of stepping as compared with standing. However, categorized stepping bout analyses revealed three key findings as follows: (1) treadmill desk users showed trends of engaging more frequently in prescribed or shorter than prescribed workday stepping bouts relative to controls (approximately 1.5 more bouts/workday) and the sit-to-stand desk group (approximately 1.2 more bouts/workday) in the short-term at M3 but did not sustain these trends after M12; (2) treadmill desk users favored accumulation of both total daily and workday long duration (ie, >30 min) stepping bouts compared with sit-to-stand desk users in the short-term after M3, which ranged from 0.1 to 0.4 more of such bouts per day, but did not sustain these trends after M12; and (3) the sit-to-stand desk group showed trends of accumulating more total daily stepping bouts of shorter than prescribed durations (ie, approximately 6 more bouts/day) relative to controls after M6, which was also not sustained after M12. Correspondingly, neither sit-to-stand nor treadmill desk groups showed short- or long-term trends of change in total daily usual stepping bout duration, and the treadmill desk group had a short-term trend after M3 of longer workday usual stepping bout durations, relative to both controls and the sit-to-stand desk group (ie, approximately 5 more min/stepping bout); this trend was only sustained long-term after M12 relative to the sit-to-stand desk group (ie, approximately 3 more min/stepping bout).

A possible explanation for the observed variability in trends of standing and stepping bout patterns between sit-to-stand and treadmill desks may be the impact of desk type on the ability to perform work. For example, when treadmill desk users were provided with feedback after M6 that they reversed positive stepping gains observed after M3, several participants in the treadmill workstation group indicated a heavy work cycle between these 2 time points as a limiting factor in their ability to walk and work on the treadmill. Participants indicated that this particular work cycle required increased multitasking involving reading, writing, and typing. This reluctance to use the treadmill desk when performing office work that likely places a higher demand on cognitive function and involves multitasking, coincides with prior evidence that treadmill desks may result in decreased performance of mouse proficiency, typing, and fine motor movement tasks [41,53]. Another study showed that these interventions may result in minor declines in overall work performance within the first 3-5 months, which may then return to or exceed baseline performance within a year of use [30]. It is likely that treadmill desk users may have failed to outlast this temporary productivity adjustment period and chose to engage in prolonged standing bouts as a compensatory behavior to decreased use of the treadmill for walking after 3 months to maintain their work productivity. Given this learning curve, future active workstation studies should consider the types of tasks office workers regularly engage in as barriers to replacing sedentary behavior with standing and stepping appropriately. Interventionists may need to design strategies to promote positive behavior change around such bouts of work during the first few months of commencing workstation use.

### Strengths and Limitations

The strengths of our study are as follows: (1) a cluster randomized controlled intervention design with a 1-year follow-up and (2) the addition of evidence on behavioral bout accumulation, which was limited to measures of central tendency for sedentary behavior bouts when using sit-to-stand desks. Our study expands the evidence base by adding a description of how sedentary, standing, and stepping bouts are accumulated across categorized bins of bout duration. This is also the first study to report the impact of treadmill desks and a head-to-head comparison of sit-to-stand and treadmill desks on behavior accumulation patterns.

Missing data are unlikely to have biased the results, given that data were determined to be missing at random and appropriately handled, but losses to follow-up may limit the generalizability of these findings because attrition rates (Multimedia Appendices 1 and 2) were higher than the trial was statistically powered to handle (ie, 20%). However, sensitivity analysis did not yield differing results. The exploratory nature of our physical behavior pattern outcome analyses is another limitation of this study, as the trial’s sample size was only powered to detect intervention effects in total daily sedentary behavior volume. Our results may thus serve as a basis for future and larger sit-to-stand and treadmill desk trials aimed at conclusively determining the effects of these workstations on physical behavior accumulation patterns among sedentary office workers. Related to sample size, the uneven allocation of participants across clusters is a limitation of this study. Another limitation of our study is that a higher ratio of enrolled women to men limits the generalizability of our findings with regard to male seated office workers. In addition, the specific contributions of the various components of the intervention on the observed trends were not discerned. Furthermore, our study did not use a preinvestigation educational approach [27,28] to assess participants’ baseline knowledge of the risks of prolonged sedentary and static standing behaviors to enhance the acceptability and responsiveness of the intended intervention.

### Conclusions

Our findings suggest that sit-to-stand desks may exert a potentially more favorable behavior change response on the accumulation patterns of physical behaviors suitable for achieving the “sit less, move more” initiative than treadmill desks. Treadmill desk users favored the accumulation of sedentary and standing bouts in prolonged durations, relative to controls and sit-to-stand desk users, which was sustained for a year. In contrast, sit-to-stand desk users showed a tendency to decrease prolonged sedentary behavior and increase short-duration standing to prescribed duration standing, which was sustained for a year. Future research on treadmill desk use needs to (1) examine whether prolonged standing bouts may be a compensatory behavior to decreased stepping and (2) study the relationship between prolonged sedentary and standing bouts to determine whether increasing prolonged sedentary behavior is a compensatory behavior to electing to stand for prolonged periods. Sit-to-stand and treadmill desks may exert a minimal impact on long-term stepping patterns, as most gains observed in our study were small and not sustained. However, sit-to-stand desk users accumulated frequent short-duration stepping over M6, which may be a beneficial behavior pattern. Future active workstation studies may require behavior change strategies that are effective in promoting more frequent bouts of movement as necessary for both (1) translating short-term stepping gains, which may be attributable to the initial novelty factor of the interventions, to long-term habitual gains and (2) limiting prolonged sedentary and standing bouts to minimize health risks.

## Appendix

Multimedia Appendix 1 Detailed study protocol.

Multimedia Appendix 2 Flow diagram of enrollment, participation, attrition, and analyses for total daily time.

Multimedia Appendix 3 Flow diagram of enrollment, participation, attrition, and analyses for workday time.

Multimedia Appendix 4 Sample sizes after losses to follow-up and activity monitor wear times by group × time point.

Multimedia Appendix 5 Intraclass correlation coefficients and significance of cluster effects by physical behavior outcome variables.

Multimedia Appendix 6 Between- and within-group comparisons of the number of daily sedentary bouts classified by bout durations over the total day and workday, adjusted for age.

Multimedia Appendix 7 Between- and within-group comparisons of the number of daily standing bouts classified by bout durations over the total day and workday, adjusted for age.

Multimedia Appendix 8 Between- and within-group comparisons of the number of daily stepping bouts classified by bout durations over the total day and workday, adjusted for age.

Multimedia Appendix 9 Baseline characteristics of aim 1 and 2 outcomes by group over the total day and workday for completers' analyses.

Multimedia Appendix 10 Between- and within-group comparisons of aim 1 and 2 outcome variables for completers' analysis.

Multimedia Appendix 11 CONSORT-eHEALTH checklist (V 1.6.1).

## Abbreviations

H: huge effect size
L: large effect size
M: medium effect size
M3: 3-month follow-up
M6: 6-month follow-up
M12: 12-month follow-up
S: small effect size
VL: very large effect size
VS: very small effect size
