# Supplementary material for: Impact of Sit-to-Stand and Treadmill Desks on Patterns of Daily Waking Physical Behaviors Among Overweight and Obese Seated Office Workers: Cluster Randomized Controlled Trial
Source: J Med Internet Res. 2023 May 16;25:e43018. doi: 10.2196/43018 (PMC10230356; doi:10.2196/43018)
Supplement: Multimedia Appendix 1 [file jmir_v25i1e43018_app1.docx]

**Detailed Protocol:**

**“Modifying the Workplace to Decrease Sedentary Behavior and Improve Health”**

**I. Background and Significance**

Prolonged sedentary behavior characterized by long bouts of sitting increases the risk for premature mortality and chronic health hazards including obesity, diabetes, cardiovascular disease, cancer and musculoskeletal disorders.[^1-8^](#_ENREF_1) This increased risk is present even among individuals who regularly engage in moderate-vigorous physical activity.[^6^](#_ENREF_6)^,^[^9^](#_ENREF_9) More than 50% of the American workforce is employed in office jobs involving prolonged sitting and are therefore at risk for these health hazards.[^10^](#_ENREF_10) Ergonomic sit-to-stand and dynamic treadmill workstations are innovative workplace interventions that allow office workers to decrease sedentary behavior and engage in light activity and standing while working.

Our preliminary 9-month trial examining the feasibility of using treadmill workstations at the workplace demonstrated that providing overweight office workers (N=12) with these dynamic workstations reduces sedentary behavior and improves overall health.[^11^](#_ENREF_11) After 3 months, participants self-selected to replace an average of 2.5 hours of sitting with 74 min of slow walking and 82 min of standing at the treadmill workstation. Workstation use declined after nine months because we did not implement any strategies to sustain this behavior change. On average, participants’ decreased weight by 2.5 + 3.9 kg and significantly reduced waist and hip circumference, total cholesterol, low-density lipoproteins and glycosylated hemoglobin. Alkhaja et al. reported that sedentary office workers using sit-to-stand workstations reduced their average sitting time by 2.3 hours during the workday and marginally improved cholesterol levels.[^12^](#_ENREF_12) In addition, Pronk et al.[^13^](#_ENREF_13) and Hedge et al.[^14^](#_ENREF_14) reported that using a sit-to-stand workstation to replace approximately 60 min of sitting with standing daily for 7 weeks significantly reduces upper back and neck pain and overall fatigue. Using sit-to-stand workstations also makes users feel more healthy, energized focused and happy.[^13^](#_ENREF_13) These data establish the feasibility of modifying the workplace using treadmill and sit-to-stand workstations to improve health.

Although prospective evidence emphasizes a reduction in sitting time to improve health, there is minimal experimental evidence demonstrating the benefits of reducing workplace sitting using treadmill and sit-to-stand workstations, which are popular alternatives to decrease workplace sitting. Only one study suggests that modifying the physical office-environment using treadmill workstations improves measures of adiposity, total cholesterol, and long-term glycemic control.[^11^](#_ENREF_11) It is not known if accumulating light intensity activity throughout the workday using treadmill workstations decreases musculoskeletal discomfort and perceived job stress. While replacing sitting with standing at work using a sit-to-stand workstation improves musculoskeletal discomfort and mood,[^13^](#_ENREF_13)^,^[^14^](#_ENREF_14) there is minimal empirical evidence demonstrating that using sit-to-stand workstations benefits cardiovascular and metabolic health. It is also necessary to compare the effects of using treadmill and sit-to-stand workstations on health because the physiological processes activated during slow walking and standing are not the same. For instance, treadmill workstations allow users to increase energy expenditure through dynamic muscular contractions, which is absent when standing (sustained static muscular contractions) at sit-to-stand workstations.[^15^](#_ENREF_15) However, sit-to-stand workstations may yield larger and sustainable decreases in workplace sedentary behavior than treadmill workstations and produce comparable health benefits over time. The abovementioned research gaps need to be examined using a robust and high-quality study methodology.

This project will provide empirical evidence on the effect and sustainability of treadmill and sit-to-stand workstations as novel ways to reduce sedentary behavior in seated office workers. A significant contribution of our project will be the development of optimal evidence-based strategies and recommendations to modify the office environment to decrease workplace sedentary behavior. The long-term benefit of decreasing workplace sedentary behavior is a significant reduction in chronic cardio-metabolic and musculoskeletal illness among employees. Employers may adopt these strategies to improve employee wellbeing and help control the growing prevalence of chronic health problems.

**II. Specific Aims**

The primary objective of this project is to determine the effects of decreasing sedentary behavior at work by at least 3hrs/day using treadmill (walking and standing) and sit-to-stand (standing only) workstations on the health of overweight office workers. Our central hypothesis is that decreasing sedentary behavior at work using these workstations will improve overall health in study participants.

**Aim 1:** **To** **conduct a 12-month, cluster-randomized trial with an intent-to-treat design to determine the effects of using treadmill and sit-to-stand workstations at the workplace on health variables in overweight office workers (N=66) with seated desk jobs.**

*Approach:* We will measure the following variables at baseline and/or after 3, 6, and 12 months: (a) anthropometrics (weight, waist and hip circumference) and body composition (b) blood-flow hemodynamics, (c) psychological affect, (d) job stress and (e) musculoskeletal discomfort. Blood biomarkers including fasting plasma glucose, HBA1C, and lipids will be measured at baseline and after 12 months.

All volunteers will attend a 30-min face-to-face information session at baseline on strategies and benefits of reducing workplace sedentary time and will be trained on office ergonomics to reduce musculoskeletal discomfort. We will then cluster randomize volunteers into a treadmill workstation (N=22), sit-to-stand workstation (N=22) and a control group (N=22). At an organizational level, department supervisors will be trained on the benefits of reducing workplace sedentary behavior and on strategies that encourage employees to sustain positive behavior change.

*Hypothesis*: We hypothesize that improvements in health variables for the sit-to-stand group will be significantly lower than the treadmill workstation group, but higher than the control group during follow-up.

**Aim 2:** **To compare the efficacies of treadmill and sit-to-stand workstations in decreasing workplace sedentary behavior.**

*Approach:* Based on our preliminary work using treadmill workstations, we will recommend a minimum use of 3 hours/day for the treadmill (walk: 2hrs and stand: 1hr) and sit-to-stand workstations. We will use accelerometers to measure time spent sitting, standing and in activity for 1-week at baseline and after 3, 6, 9 and 12 months.

*Hypothesis 2A*: Decrease in sedentary behavior will be greatest in the sit-to-stand group and lowest in the control group.

*Hypothesis 2B*: Based on our preliminary work, we postulate that decreasing workplace sedentary behavior in the intervention groups will not result in a compensatory increase in sedentary behavior outside the workplace.

**III. Subject Selection**

a) Inclusion and Exclusion criteria

1. Participants have a body mass index greater than 25 kg/m^2^
2. Do not engage in any structured physical activity for more than 2 days/week
3. Are employed in a seated desk job
4. Are free of limitations that prevent walking and standing in bouts lasting 40 to 60 min.
5. Women who are pregnant.

b) Source of subjects and Recruitment methods

Sixty-six participants will be recruited from the Massachusetts General Hospital and Northeastern University. Potential departments at Massachusetts General Hospital include The Cancer Center (protocol office and call center), Professional Billing Office, Materials Management Customer Service, Health and Information Services, Radiology Service Center, and Research Management. Departments at Northeastern University include Speech-Language Pathology Clinic, Department of Communication Sciences and Disorders, Bouve College Office of Research, The John O'Bryant African American Institute, and Northeastern University Humanities Center. These departments were previously identified as potential sites after informal communication about the study with the department heads and human resource management.

**IV. Subject Enrollment**

a) Methods of enrollment, including procedures for patient registration and/or randomization

A trained project manager will meet potential participants as a group during department meetings and verbally explain study procedures and inclusion criteria. Eligibility will be assessed based on self-report and measured height and weight. If employees from a department express interest in the study, the department will be cluster randomized into either the treadmill workstation, sit-stand workstation, or control group after completing informed consent procedures. Twenty-two volunteers will be randomly selected from each department group. In the event of insufficient volunteers in a group, the project manager will approach other departments to solicit study participation. Our recruitment target will include at least 50% women and 20% minorities, respectively. However, the study design may limit our ability to effectively recruit these two categories of participants.

b) Procedures for obtaining informed consent (including timing of consent process)

Informed consent process will be conducted during the lunch hour or a convenient time for the participant after work hours. This visit will be conducted at the participant’s physical place of work or in a private setting (if desired by the participant) on the premises of the institution. The informed consent process will be conducted by a trained project manager who will give a detailed verbal description of the study to the participants. These details will include a description of the study and complete disclosure of the various measurements and procedures that will be performed during the course of the study including the risks and discomforts as well as potential benefits associated with participation. They will then be provided with a hard copy of the informed consent form to read. Volunteers will be allowed to ask questions and clarify all their doubts during the consent process. Volunteers will be free to sign the informed consent during this visit or take up to 2 days to make a decision on study participation. They will be informed that they are free to terminate study participation at any point during the study. After they make a decision, the participants will sign the informed consent document and return it to the project manager. A copy of the signed and dated informed consent form will be given to the subject. This visit will take approximately 30 minutes to complete. If participants agree to participate, they will also complete a self-report health history questionnaire that will gather information on any pre-existing conditions related to cardio-vascular, metabolic and musculoskeletal disease. On this questionnaire, participants will also report if they are on any medications and the current dosage. In addition, participants will also complete a brief questionnaire to determine their habitual physical activity levels.

c) Treatment assignment and randomization

We will do a cluster randomization of various departments mentioned above into the control and 2 intervention groups. If employees from a department express interest in the study, the department will be cluster randomized into either the treadmill workstation, sit-stand workstation, or control group. Twenty-two volunteers will be randomly selected from each department group. In the event of insufficient volunteers in a group, the project manager will approach other departments within the hospital to solicit study participation.

**V. Study Procedures**

a) Training to use workstations and related measurements

Prior to randomization, all 66 participants will receive a 30 min face-to-face counseling session with a trained researcher on evidence-based benefits of reducing sedentary behavior and will be taught various strategies to decrease workplace sitting (e.g., taking stretch breaks, walking to a colleague’s desk instead of using the phone). We will provide individual onsite training to department-heads/supervisors in the intervention groups on the benefits and strategies to decrease sedentary behavior at work and on techniques to provide verbal encouragement to employees. Department-heads/supervisors will use these techniques to reinforce the importance of decreasing sedentary behavior at regular department meetings during the intervention. Individual participant and department-head/supervisor training sessions will be repeated after 3, 6 and 9 months.

Prior to installing the workstations in the offices of the two intervention groups, participants will be trained on correctly using the workstation. Training will be based on general recommendations from the Occupational Safety and Health Administration and will consist of information on selecting the appropriate height of the work surface and maintaining good posture while working in the sitting and standing positions. These recommendations will be individualized for participants by measuring the height of the tabletop and the horizontal position of the keyboard, mouse, and monitor and their angles relative to the horizontal plane. Based on qualitative feedback from our preliminary study, we will make the following recommendations to the treadmill workstation group: “(1) Acclimate to walking on the treadmill during the first week at a speed between 0.7 to 1.0mph, (2) walk and stand for short bouts of 10-min during acclimation, (3) after acclimation, walk at a speed between 1.0 and 2.0mph as this range allows you to simultaneously perform work and minimally affects work performance and (4) after acclimation, accumulate periods of walking and standing during the course of the day in bouts lasting between 10 and 30 min.” Participants in the treadmill workstation group will be recommend to accumulate at least 2 hours of walking and 1 hour of standing/day. This 3-hour recommendation is based on previous findings. Similarly, we will recommend participants to acclimate themselves to standing during the first week of workstation use and accumulate a minimum of 3 hours of standing per day in bouts lasting 10 to 30 min after acclimation. We will recommend the control group to engage in three 10-min bouts of walking during the workday: one during each of the morning and afternoon sessions of work and a third during the lunch break.

b) Study visits and parameters to be measured

The participant will complete measures 1 to 5 in a lab and measures 6 to 8 at home within 1-week of completing the former. Several of these measures will be recorded on a hard-copy data collection sheet.

1. **Anthropometrics (Baseline, Months 3, 6, and 12):** Anthropometrics will include height, weight, and waist and hip circumference measures. Weight and height will be measured using a standard weighing scale and stadiometer with the participant clothed in a hospital gown or light weight clothing, respectively. Waist and hip circumferences will be measured in the standing position using a spring gauge tensiometer Gullick tape. Waist circumference will be made at the narrowest part of the torso and hip circumference will be measured at the widest portion of the buttocks. Three measurements will be made at each site.
2. **Blood Draw (Baseline and Month 12):** A certified phlebotomist will draw approximately 15 ml of blood from a vein at the antecubital region of the elbow. This sample will be analyzed for fasting plasma glucose, Glycosylated hemoglobin (HBA1C), insulin, and lipids.
3. **Body Composition (Baseline, Months 3 and 12):** Body composition will be measured using dual energy X-ray absorptiometry (DEXA) (Lunar DXA, GE HealthCare, Fairfield, CT.). DEXA is an enhanced form of X-ray technology and is a painless test. The effective radiation dose from this procedure is about the same as the average person receives from background radiation in one day. A certified technician will conduct this test.

During the test, participants will remove all metallic objects from their person and clothing and lay down on the testing bed. The test will last for about 5 minutes and during the test, a scanner will pass over the body. Body composition variables will be derived using proprietary software.

1. **Resting heart rate and blood pressure (Baseline, Months 3, 6, and 12):** These will be measured after 10 minutes of seated rest and repeated thrice with a two-minute break between measurements. Heart rate will be measured using the palpation technique at the radial artery of the wrist or using a Polar heart watch. Trained personnel will measure resting blood pressure using a manually inflatable blood pressure cuff and monitor.
2. **Hemodynamics (Baseline, Months 3, 6, and 12):** For the popliteal artery, we will measure shear rate (popliteal hyperemic response) using an Ultrasound Doppler system with the subject lying down. Hyperemic responses will be measured after a 5-min arterial occlusion using cuff inflation at 250 mmHg. For hyperemic shear rate, peak systolic velocity will be obtained from the first 15 seconds following cuff release and peak diameter change from baseline observed continuously in the 45-90 second post-occlusion period. These measurements will be made six times and shear rate will be calculated using Poiseuille’s Law.
3. **Affect and stress (Baseline, Months 3, 6, and 12):** Participants will be requested to complete a brief Positive and Negative Affect Schedule to measure emotional status. The questionnaire consists of 20 words describing different emotional states and participants will use a scale (range: 1 =slightly and 5 = very much) to indicate how much they experienced each emotion during the past week. Participants will report job stress on the modified National Institute of Occupational Safety and Health Generic Job Stress Questionnaire that examines psychological demands, decision latitude and support at work. This questionnaire contains 68 question that need to be rated on a Likert scale ranging between 1 (strongly disagree) and 5 (strongly agree)
4. **Musculoskeletal discomfort symptoms (Baseline, Months 3, 6, and 12):** Participants will indicate on a questionnaire containing a pictograph of the human body if they have experienced any pain or discomfort in the neck, shoulder, lower back, wrist or forearm, knee, and ankle or feet during the last 3 months. They will be requested to rate the pain using a scale ranging from 0 (no pain) to 4 extreme pain). They will then rate the magnitude of the pain on a Likert scale ranging between 0 to 10 (0= no pain, 10= worst pain imaginable)
5. **Dietary Intake (Baseline, Months 3, 6, and 12):** Participants will report frequency and portion size of foods consumed during the past 3 months from a list of 70 commonly consumed food items on a paper pencil questionnaire.
6. **Workstation related measures (Baseline):** Workstation measures will be made within 1 to 2 weeks of installation in participant offices. These include, the height of the tabletop and the horizontal position of the keyboard, mouse, and monitor and their angles relative to the horizontal plane.

b) Drugs to be used

N/A

c) Devices to be used

**Activity monitoring:** This measurement will be conducted for a period of 1 week. Participants will be asked to wear 2 small activity monitors (53 x 35 x 7 mm, weight=15 grams), one on the right thigh and the other on the torso (chest or abdomen). The device will be attached to the skin using medical grade, hypoallergenic tape or soft elastic bands. We will measure time spent at work and outside the workplace for one week using radio frequency identification technology (RFID). A thin RFID sticker tag (120 mm x 44 mm x 0.4 mm) will be wrapped around the activity monitor on the torso. This will record every time a participant enters and leaves their place of work on an RFID reader installed in the building. During this 1-week period, participants will record the times they wore and removed the monitors and the times they arrived and left their workplace. Based on the data collected using these devices, participants will be provided with retrospective feedback every 3 months on their activity behavior.

d) Procedures/surgical interventions, etc.

N/A

VI. Biostatistical Analyses

a) Specific data variables being collected during the study (e.g., data collection sheets)

Please see attached data collection forms:

1. Data collection sheet
2. Monitor log
3. Health History Questionnaire
4. Brief Block Food Questionnaire
5. Ergonomic measurements
6. Modified National Institute of Occupational Safety and Health Generic Job Stress Questionnaire
7. Positive and Negative Affect Schedule
8. Musculoskeletal Discomfort questionnaire

b) Statistical methods

Aim1: Hypothesis testing will entail an intent-to-treat two-tailed design with a nominal type I error of 0.05.  Preliminary descriptive analyses will explore observed differences in subject characteristics that may be related to study outcomes between the three groups. Variables with notable differences will be included in multivariable models. Mixed linear regression models will be used to compare differences between groups across all measurement points.  These models will include variables identified in the preliminary analyses as potential confounders.

Aim2: We will use mixed linear regression models to examine within and between-group differences in the total time spent in sedentary behavior, sitting, standing, and walking while at work and outside the workplace.

c) Power analyses

Sample size calculations are based on body weight change among participants (N=12) in our preliminary study.[^11^](#_ENREF_11) Our current study will have 22 participants in each of the three study arms. This will provide sufficient power to detect an effect size of 0.89 between each of the groups where the effect size = Δ/s (Δ: mean difference between two groups; s: common standard deviation). A standard deviation of 3.9 kg for weight observed in our pilot study corresponds to a mean detectable difference of 3.5 kg between groups. Although we anticipate minimal clustering within the departments at the study site, the power estimates presented above are based on an effective sample size of 60 subjects reflecting an intra-class correlation coefficient of 0.01. Comparisons between the most effective intervention and the control group will have substantially greater power reflecting larger differences in mean measurements.

**VII. Risks and Discomforts**

a) Common

i) Physical risks associated with the blood draw may include minor temporary discomfort, pain, and/or bruising, and result in an infection on rare occasions.

ii) Participants may also feel minor discomfort at the thigh when the blood pressure cuff is inflated to measure shear rate.

iii) There is a rare risk of a participant falling off the treadmill belt during slow walking. Height of the floor around the treadmill will be elevated to that of the treadmill belt to minimize trip hazard.

iv) The amount of radiation exposure received from the DEXA scan in this study is below the levels that may result in a significant risk of harmful effects. The potential increase the risk of other adverse health consequences due to low level radiation exposure from the DEXA scan is too small to be estimated accurately. As a result of your participation in this study, you will be exposed to radiation from the DEXA scan procedure to measure your bone density.   The amount of radiation to which you will be exposed is less than 3% of the annual background radiation everyone is exposed to each year from the earth and sky.

b) Uncommon

Participant recruitment for this study is not confidential. Thus, co-workers will know if a person is participating in one of the two intervention arms. It is possible that a study subject could experience some social discomfort since one of the recruitment criteria will indicate that he or she has a body mass index of least 25 kg/m^2^.

**VIII) Potential Benefits**

a) Potential benefits to participants

Participants in this study may experience no direct benefit from participating in the study. It is hoped that those who are assigned to the two interventions may directly benefit from a reduction in sedentary behavior and improve overall health. Replacing sitting with standing and walking may improve cardio-metabolic variables and decrease musculoskeletal discomfort and stress among participants.

b) Potential benefits to society

In the future, the results of this study may benefit other employees by providing evidence that justifies the utility of modifying the workspace using treadmill and sit-stand workstations to improve health in overweight office workers.

**IX) Monitoring and Quality Assurance**

a) Independent monitoring of source data

Data will be collected at baseline and after 3, 6, and 12 months. Regular monitoring of the safety of data and documentation collected during these visits will be conducted after procedures are completed for 10 participants. The monitoring will be completed by the project manager under the supervision of the principal investigator of the study at Northeastern University.

b) Safety monitoring

A trained research staff member will meet with study participants twice a month and examine workstation function and set up. The staff member will also directly enquire if participants have any concerns regarding being part of the study. The participant can terminate participation in this study at any time if they do not wish to continue being a part of the study. There are no specific rules for stopping the study other than the occurrence of an adverse event.

c) Outcomes monitoring

Regular monitoring of the safety and efficacy of data and documentation collected during the visits when outcome variables are measured will be conducted after the procedures are completed for 10 participants. The monitoring will be completed by the project manager under the supervision of the principal investigator of the study at Northeastern University. Participation in this study can be terminated by the participant at any time if they do not wish to continue being a part of the study. There are no specific rules for stopping the study.

d) Adverse event reporting guidelines

Study participants will be monitored for the occurrence of adverse events. Adverse event reporting will be in accordance with the Partners Healthcare policies and guidelines. These reports will include a description of all events, participant’s condition after the event, an estimate of the extent of injury and potential strategies to prevent future occurrences. The principal investigator will classify the relationship of the study protocol to the event. The principal investigator for the study and/or the site PI will be responsible for reporting serious adverse events (death, life threatening, new, serious or permanent disability) to the Institutional Review Board. Unanticipated problems involving risks to subjects or others including adverse events will be reported to the IRB within 5 working days/7 calendar days of the date the investigator first becomes aware of the problem, which is in accordance with Partners Health Care IRB reporting guidelines.

**X. References**

1. Abasolo L, Lajas C, Leon L, et al. Prognostic factors for long-term work disability due to musculoskeletal disorders. *Rheumatology international.* 2011.

2. Bankoski A, Harris TB, McClain JJ, et al. Sedentary activity associated with metabolic syndrome independent of physical activity. *Diabetes care.* 2011;34(2):497-503.

3. Gardiner PA, Healy GN, Eakin EG, et al. Associations between television viewing time and overall sitting time with the metabolic syndrome in older men and women: the Australian Diabetes, Obesity and Lifestyle study. *Journal of the American Geriatrics Society.* 2011;59(5):788-796.

4. Healy B, Levin E, Perrin K, Weatherall M, Beasley R. Prolonged work-and computer-related seated immobility and risk of venous thromboembolism. *JRSM.* 2010;103(11):447-454.

5. Healy GN, Dunstan DW, Salmon J, et al. Objectively measured light-intensity physical activity is independently associated with 2-h plasma glucose. *Diabetes care.* 2007;30(6):1384-1389.

6. Katzmarzyk PT, Church TS, Craig CL, Bouchard C. Sitting time and mortality from all causes, cardiovascular disease, and cancer. *Medicine and science in sports and exercise.* 2009;41(5):998-1005.

7. Mummery WK, Schofield GM, Steele R, Eakin EG, Brown WJ. Occupational sitting time and overweight and obesity in Australian workers. *American journal of preventive medicine.* 2005;29(2):91-97.

8. Wijndaele K, Healy GN, Dunstan DW, et al. Increased cardiometabolic risk is associated with increased TV viewing time. *Medicine and science in sports and exercise.* 2010;42(8):1511-1518.

9. Dunstan DW, Barr EL, Healy GN, et al. Television viewing time and mortality: the Australian Diabetes, Obesity and Lifestyle Study (AusDiab). *Circulation.* 2010;121(3):384-391.

10. U.S. Census Bureau. U.S. Department of Commerce. <http://www.census.gov/hhes/computer/>. Updated 07-11-2012. Accessed 10-15-2012, 2012.

11. John D, Thompson DL, Raynor H, Bielak K, Rider B, Bassett DR. Treadmill workstations: a worksite physical activity intervention in overweight and obese office workers. *Journal of physical activity & health.* 2011;8(8):1034-1043.

12. Alkhajah TA, Reeves MM, Eakin EG, Winkler EA, Owen N, Healy GN. Sit-stand workstations: a pilot intervention to reduce office sitting time. *American journal of preventive medicine.* 2012;43(3):298-303.

13. Pronk NP, Katz AS, Lowry M, Payfer JR. Reducing Occupational Sitting Time and Improving Worker Health: The Take-a-Stand Project, 2011. *Preventing chronic disease.* 2012;9:E154.

14. Hedge A, Ray EJ. Effects of an Electronic Height-Adjustable Worksurface on Computer Worker Musculoskeletal Discomfort and Productivity. Paper presented at: Proceedings of the Human Factors and Ergonomics Society Annual Meeting2004.

15. Levine JA, Miller JM. The energy expenditure of using a "walk-and-work" desk for office workers with obesity. *British journal of sports medicine.* 2007;41(9):558-561.
