# Supplementary material for: Impact of Sit-to-Stand and Treadmill Desks on Patterns of Daily Waking Physical Behaviors Among Overweight and Obese Seated Office Workers: Cluster Randomized Controlled Trial
Source: J Med Internet Res. 2023 May 16;25:e43018. doi: 10.2196/43018 (PMC10230356; doi:10.2196/43018)
Supplement: Multimedia Appendix 2 [file jmir_v25i1e43018_app2.docx]

Supplemental Figure 1. Flow diagram of enrollment, participation, attrition, and analyses for total-daily time
