# Supplementary material for: Impact of Sit-to-Stand and Treadmill Desks on Patterns of Daily Waking Physical Behaviors Among Overweight and Obese Seated Office Workers: Cluster Randomized Controlled Trial
Source: J Med Internet Res. 2023 May 16;25:e43018. doi: 10.2196/43018 (PMC10230356; doi:10.2196/43018)
Supplement: Multimedia Appendix 4 [file jmir_v25i1e43018_app4.docx]

Supplemental Table 1. Sample sizes after losses to follow-up and activity monitor weartimes by group x timepoint
