# Supplementary material for: Impact of Sit-to-Stand and Treadmill Desks on Patterns of Daily Waking Physical Behaviors Among Overweight and Obese Seated Office Workers: Cluster Randomized Controlled Trial
Source: J Med Internet Res. 2023 May 16;25:e43018. doi: 10.2196/43018 (PMC10230356; doi:10.2196/43018)
Supplement: Multimedia Appendix 5 [file jmir_v25i1e43018_app5.docx]

Supplemental Table 2. Intraclass Correlation Coefficients (ICC) and significance of cluster effects by physical behavior outcome variables

Key: No. = number, significant *P*<.05

Cluster sizes: seated-desk control (8 clusters; N’s: 7, 3, 4, 2, 1, 1, 2, 1); sit-to-stand desk (9 clusters: N’s: 4, 8, 2, 2, 2, 2, 1, 1, 1); and treadmill desk (7 clusters; N’s: 1, 3, 11, 1, 1, 4, 1).
