# Supplementary material for: Impact of Sit-to-Stand and Treadmill Desks on Patterns of Daily Waking Physical Behaviors Among Overweight and Obese Seated Office Workers: Cluster Randomized Controlled Trial
Source: J Med Internet Res. 2023 May 16;25:e43018. doi: 10.2196/43018 (PMC10230356; doi:10.2196/43018)
Supplement: Multimedia Appendix 7 [file jmir_v25i1e43018_app7.docx]

Supplemental Table 4. Between- and within-group comparisons of the number of daily standing bouts classified by bout durations over the total-day and workday, adjusted for age

Key: No. = number, M3= month-3 follow-up, M6= month-6 follow-up, M12= month-12 follow-up, VS= very small effect size, S= small effect size, M= medium effect side, L= large effect size,

** = effect trend (i.e., unidirectional 95% CI’s not overlapping null value),

⟟ = adjusted for baseline

Sample sizes after losses to follow-up: Baseline N = 66 (21 controls, 23 sit-to-stand desk, 22 treadmill desk), M3 N = 58 (15 controls, 21 sit-to-stand desk, 22 treadmill desk), M6 N = 53 (14 controls, 20 sit-to-stand desk, 19 treadmill desk), and M12 N = 58 (18 controls, 20 sit-to-stand desk, 20 treadmill desk)
