# Supplementary material for: Impact of Sit-to-Stand and Treadmill Desks on Patterns of Daily Waking Physical Behaviors Among Overweight and Obese Seated Office Workers: Cluster Randomized Controlled Trial
Source: J Med Internet Res. 2023 May 16;25:e43018. doi: 10.2196/43018 (PMC10230356; doi:10.2196/43018)
Supplement: Multimedia Appendix 10 [file jmir_v25i1e43018_app10.docx]

Supplemental Table 7. Between and within group comparisons of aim 1 and 2 outcome variables for completer’s analysis.

Key: M12= month-12 follow-up, VS= very small effect size, S= small effect size, M= medium effect side, L= large effect size, ** = effect trend (i.e., unidirectional 95% CI’s not overlapping null value).

Baseline to M12 N: total-day= 42 (13 controls, 13 sit-to-stand desk, 16 treadmill desk), workday= 35 (10 controls, 12 sit-to-stand desk, 13 treadmill desk).
