# Supplementary material for: Impact of Sit-to-Stand and Treadmill Desks on Patterns of Daily Waking Physical Behaviors Among Overweight and Obese Seated Office Workers: Cluster Randomized Controlled Trial
Source: J Med Internet Res. 2023 May 16;25:e43018. doi: 10.2196/43018 (PMC10230356; doi:10.2196/43018)
Supplement: Multimedia Appendix 11 [file jmir_v25i1e43018_app11.pdf]

CONSORT-EHEALTH (V 1.6.1) - Submission/Publication Form

The CONSORT-EHEALTH checklist is intended for authors of randomized trials evaluating web-based and Internet-based applications/interventions, including mobile interventions, electronic games (incl multiplayer games), social media, certain telehealth applications, and other interactive and/or networked electronic applications. Some of the items (e.g. all subitems under item 5 - description of the intervention) may also be applicable for other study designs.

The goal of the CONSORT EHEALTH checklist and guideline is to be
a) a guide for reporting for authors of RCTs,
b) to form a basis for appraisal of an ehealth trial (in terms of validity)

CONSORT-EHEALTH items/subitems are MANDATORY reporting items for studies published in the Journal of Medical Internet Research and other journals / scientific societies endorsing the checklist.

Items numbered 1., 2., 3., 4a., 4b etc are original CONSORT or CONSORT-NPT (non-pharmacologic treatment) items.
Items with Roman numerals (i., ii, iii, iv etc.) are CONSORT-EHEALTH extensions/clarifications.

As the CONSORT-EHEALTH checklist is still considered in a formative stage, we would ask that you also RATE ON A SCALE OF 1-5 how important/useful you feel each item is FOR THE PURPOSE OF THE CHECKLIST and reporting guideline (optional).

Mandatory reporting items are marked with a red *.
In the textboxes, either copy & paste the relevant sections from your manuscript into this form - please include any quotes from your manuscript in QUOTATION MARKS,
or answer directly by providing additional information not in the manuscript, or elaborating on why the item was not relevant for this study.

YOUR ANSWERS WILL BE PUBLISHED AS A SUPPLEMENTARY FILE TO YOUR PUBLICATION IN JMIR AND ARE CONSIDERED PART OF YOUR PUBLICATION (IF ACCEPTED).
Please fill in these questions diligently. Information will not be copyedited, so please use proper spelling and grammar, use correct capitalization, and avoid abbreviations.

DO NOT FORGET TO SAVE AS PDF _AND_ CLICK THE SUBMIT BUTTON SO YOUR ANSWERS ARE IN OUR DATABASE !!!

Citation Suggestion (if you append the pdf as Appendix we suggest to cite this paper in the caption):
Eysenbach G, CONSORT-EHEALTH Group

CONSORT-EHEALTH: Improving and Standardizing Evaluation Reports of Web-based and Mobile Health Interventions
J Med Internet Res 2011;13(4):e126
URL: http://www.jmir.org/2011/4/e126/
doi: 10.2196/jmir.1923
PMID: 22209829

diego.arguello.da@gmail.com (not shared) Switch account

Draft not saved

* Required

Your name *

First Last

Diego Arguello

Primary Affiliation (short), City, Country *

University of Toronto, Toronto, Canada

Northeastern University, Boston, MA

Your e-mail address *

abc@gmail.com

arguello.d@northeastern.edu

Title of your manuscript *

Provide the (draft) title of your manuscript.

Impact of a Sit-to-Stand and Treadmill Desk Cluster-Randomized Controlled Trial on Patterns of Daily Waking Physical Behaviors Among Overweight and Obese Seated Office Workers

Name of your App/Software/Intervention *

If there is a short and a long/alternate name, write the short name first and add the long name in brackets.

Modifying the Workplace to Decrease Sedenta

Evaluated Version (if any)

e.g. "V1", "Release 2017-03-01", "Version 2.0.27913"

V1.6.1

Language(s) *

What language is the intervention/app in? If multiple languages are available, separate by comma (e.g. "English, French")

English

URL of your Intervention Website or App

e.g. a direct link to the mobile app on app in appstore (itunes, Google Play), or URL of the website. If the intervention is a DVD or hardware, you can also link to an Amazon page.

Your answer

URL of an image/screenshot (optional)

Your answer

Accessibility *

Can an enduser access the intervention presently?

- ◯ access is free and open
- ◯ access only for special usergroups, not open
- ◯ access is open to everyone, but requires payment/subscription/in-app purchases
- ◯ app/intervention no longer accessible
- ◉ Other: Access was given only to a specific study population in the form of sit

Primary Medical Indication/Disease/Condition *

e.g. "Stress", "Diabetes", or define the target group in brackets after the condition, e.g. "Autism (Parents of children with)", "Alzheimers (Informal Caregivers of)"

Overweight or obese sedentary office workers

Primary Outcomes measured in trial *

comma-separated list of primary outcomes reported in the trial

Sedentary behavior, and health outcomes (i.e.,

Secondary/other outcomes

Are there any other outcomes the intervention is expected to affect?

Upright physical behaviors (i.e., standing and stepping)

Recommended "Dose" *

What do the instructions for users say on how often the app should be used?

- ○ Approximately Daily
- ○ Approximately Weekly
- ○ Approximately Monthly
- ○ Approximately Yearly
- ○ "as needed"
- ◉ Other: " Treadmill and sit-to-stand desk workers were recommended to repla

Approx. Percentage of Users (starters) still using the app as recommended after 3 months *

- ○ unknown / not evaluated
- ○ 0-10%
- ○ 11-20%
- ○ 21-30%
- ○ 31-40%
- ○ 41-50%
- ○ 51-60%
- ○ 61-70%
- ○ 71%-80%
- ○ 81-90%
- ○ 91-100%
- ◉ Other: Approximately 96% of sit-to-stand and treadmill desk users were still [illegible]

Overall, was the app/intervention effective? *

- ○ yes: all primary outcomes were significantly better in intervention group vs control
- ◉ partly: SOME primary outcomes were significantly better in intervention group vs control
- ○ no statistically significant difference between control and intervention
- ○ potentially harmful: control was significantly better than intervention in one or more outcomes
- ○ inconclusive: more research is needed
- ○ Other:

Article Preparation Status/Stage *

At which stage in your article preparation are you currently (at the time you fill in this form)

- ○ not submitted yet - in early draft status
- ◉ not submitted yet - in late draft status, just before submission
- ○ submitted to a journal but not reviewed yet
- ○ submitted to a journal and after receiving initial reviewer comments
- ○ submitted to a journal and accepted, but not published yet
- ○ published
- ○ Other:

Journal *

If you already know where you will submit this paper (or if it is already submitted), please provide the journal name (if it is not JMIR, provide the journal name under "other")

- ○ not submitted yet / unclear where I will submit this
- ◉ Journal of Medical Internet Research (JMIR)
- ○ JMIR mHealth and UHealth
- ○ JMIR Serious Games
- ○ JMIR Mental Health
- ○ JMIR Public Health
- ○ JMIR Formative Research
- ○ Other JMIR sister journal
- ○ Other:

Is this a full powered effectiveness trial or a pilot/feasibility trial? *

- ( ) Pilot/feasibility
- (•) Fully powered

Manuscript tracking number *

If this is a JMIR submission, please provide the manuscript tracking number under "other" (The ms tracking number can be found in the submission acknowledgement email, or when you login as author in JMIR. If the paper is already published in JMIR, then the ms tracking number is the four-digit number at the end of the DOI, to be found at the bottom of each published article in JMIR)

- (•) no ms number (yet) / not (yet) submitted to / published in JMIR
- ( ) Other:

TITLE AND ABSTRACT

1a) TITLE: Identification as a randomized trial in the title

1a) Does your paper address CONSORT item 1a? *

I.e does the title contain the phrase "Randomized Controlled Trial"? (if not, explain the reason under "other")

- (•) yes
- ( ) Other:

1a-i) Identify the mode of delivery in the title

Identify the mode of delivery. Preferably use “web-based” and/or “mobile” and/or “electronic game” in the title. Avoid ambiguous terms like “online”, “virtual”, “interactive”. Use “Internet-based” only if Intervention includes non-web-based Internet components (e.g. email), use “computer-based” or “electronic” only if offline products are used. Use “virtual” only in the context of “virtual reality” (3-D worlds). Use “online” only in the context of “online support groups”. Complement or substitute product names with broader terms for the class of products (such as “mobile” or “smart phone” instead of “iphone”), especially if the application runs on different platforms.

	1	2	3	4	5	
subitem not at all important	○	○	○	○	○	essential

Does your paper address subitem 1a-i? *

Copy and paste relevant sections from manuscript title (include quotes in quotation marks "like this" to indicate direct quotes from your manuscript), or elaborate on this item by providing additional information not in the ms, or briefly explain why the item is not applicable/relevant for your study

Subitem 1a-i is not applicable to our study, as our intervention was not web-, mobile- nor computer-based, but rather focused on the installation of new technologies (sit-to-stand and treadmill desks) as an environmental intervention to reduce sedentary behavior at the workplace.

1a-ii) Non-web-based components or important co-interventions in title

Mention non-web-based components or important co-interventions in title, if any (e.g., “with telephone support”).

	1	2	3	4	5	
subitem not at all important	○	○	○	○	○	essential

Does your paper address subitem 1a-ii?

Copy and paste relevant sections from manuscript title (include quotes in quotation marks "like this" to indicate direct quotes from your manuscript), or elaborate on this item by providing additional information not in the ms, or briefly explain why the item is not applicable/relevant for your study

"Impact of a Sit-to-Stand and Treadmill Desk"

1a-iii) Primary condition or target group in the title

Mention primary condition or target group in the title, if any (e.g., "for children with Type I Diabetes") Example: A Web-based and Mobile Intervention with Telephone Support for Children with Type I Diabetes: Randomized Controlled Trial

	1	2	3	4	5	
subitem not at all important	○	○	○	○	○	essential

Does your paper address subitem 1a-iii? *

Copy and paste relevant sections from manuscript title (include quotes in quotation marks "like this" to indicate direct quotes from your manuscript), or elaborate on this item by providing additional information not in the ms, or briefly explain why the item is not applicable/relevant for your study

"Among Overweight and Obese Seated Office Workers"

1b) ABSTRACT: Structured summary of trial design, methods, results, and conclusions

NPT extension: Description of experimental treatment, comparator, care providers, centers, and blinding status.

1b-i) Key features/functionalities/components of the intervention and comparator in the METHODS section of the ABSTRACT

Mention key features/functionalities/components of the intervention and comparator in the abstract. If possible, also mention theories and principles used for designing the site. Keep in mind the needs of systematic reviewers and indexers by including important synonyms. (Note: Only report in the abstract what the main paper is reporting. If this information is missing from the main body of text, consider adding it)

	1	2	3	4	5	
subitem not at all important	○	○	○	○	○	essential

Does your paper address subitem 1b-i? *

Copy and paste relevant sections from the manuscript abstract (include quotes in quotation marks "like this" to indicate direct quotes from your manuscript), or elaborate on this item by providing additional information not in the ms, or briefly explain why the item is not applicable/relevant for your study

In the abstract we stated that this is a "multi-component intervention", which was described in detail within the method's "Intervention" subsection of the manuscript. We also denoted the comparator by stating: "Sixty-six office workers were cluster-randomized into a seated-desk control (n = 21; 8 clusters), sit-to-stand desk (n = 23; 9 clusters), or treadmill desk (n = 22; 7 clusters) group."

1b-ii) Level of human involvement in the METHODS section of the ABSTRACT

Clarify the level of human involvement in the abstract, e.g., use phrases like “fully automated” vs. “therapist/nurse/care provider/physician-assisted” (mention number and expertise of providers involved, if any). (Note: Only report in the abstract what the main paper is reporting. If this information is missing from the main body of text, consider adding it)

	1	2	3	4	5	
subitem not at all important	○	○	○	○	○	essential

Does your paper address subitem 1b-ii?

Copy and paste relevant sections from the manuscript abstract (include quotes in quotation marks "like this" to indicate direct quotes from your manuscript), or elaborate on this item by providing additional information not in the ms, or briefly explain why the item is not applicable/relevant for your study

This is not applicable to the main component of the intervention, which consisted of an environmental modification in the workplace, but we stated that this component of the intervention was supplemented with participants receiving "periodic feedback on their physical behaviors".

1b-iii) Open vs. closed, web-based (self-assessment) vs. face-to-face assessments in the METHODS section of the ABSTRACT

Mention how participants were recruited (online vs. offline), e.g., from an open access website or from a clinic or a closed online user group (closed usergroup trial), and clarify if this was a purely web-based trial, or there were face-to-face components (as part of the intervention or for assessment). Clearly say if outcomes were self-assessed through questionnaires (as common in web-based trials). Note: In traditional offline trials, an open trial (open-label trial) is a type of clinical trial in which both the researchers and participants know which treatment is being administered. To avoid confusion, use “blinded” or “unblinded” to indicated the level of blinding instead of “open”, as “open” in web-based trials usually refers to “open access” (i.e. participants can self-enrol). (Note: Only report in the abstract what the main paper is reporting. If this information is missing from the main body of text, consider adding it)

	1	2	3	4	5	
subitem not at all important	○	○	○	○	○	essential

Does your paper address subitem 1b-iii?

Copy and paste relevant sections from the manuscript abstract (include quotes in quotation marks "like this" to indicate direct quotes from your manuscript), or elaborate on this item by providing additional information not in the ms, or briefly explain why the item is not applicable/relevant for your study

For conciseness in the abstract we stated that this is a "multicomponent intervention" to point readers to the detailed methods where we stated: "Enrolled participants received initial face-to-face counseling sessions on the benefits of sitting less, standing and moving more. Supervisors of those participants in the treadmill and sit-to-stand desk groups received initial on-site training on the benefits of decreasing sedentary behavior in the workplace and on strategies to encourage participant engagement in the intervention, which they did so at regular department meetings. Participant counseling and supervisor trainings were repeated every three months for the treadmill and sit-to-stand desk groups, and these participants were also given feedback on their measured physical behaviors (described below) during these sessions. "

1b-iv) RESULTS section in abstract must contain use data

Report number of participants enrolled/assessed in each group, the use/uptake of the intervention (e.g., attrition/adherence metrics, use over time, number of logins etc.), in addition to primary/secondary outcomes. (Note: Only report in the abstract what the main paper is reporting. If this information is missing from the main body of text, consider adding it)

	1	2	3	4	5	
subitem not at all important	○	○	○	○	○	essential

Does your paper address subitem 1b-iv?

Copy and paste relevant sections from the manuscript abstract (include quotes in quotation marks "like this" to indicate direct quotes from your manuscript), or elaborate on this item by providing additional information not in the ms, or briefly explain why the item is not applicable/relevant for your study

For conciseness, we chose to report the initial enrollment for each study arm in the abstract, but left attrition and adherence metrics to the manuscript and supplementary materials as there was too much data to report on these metrics given that our study design consisted of three follow-ups over a year.

1b-v) CONCLUSIONS/DISCUSSION in abstract for negative trials

Conclusions/Discussions in abstract for negative trials: Discuss the primary outcome - if the trial is negative (primary outcome not changed), and the intervention was not used, discuss whether negative results are attributable to lack of uptake and discuss reasons. (Note: Only report in the abstract what the main paper is reporting. If this information is missing from the main body of text, consider adding it)

	1	2	3	4	5	
subitem not at all important	○	○	○	○	○	essential

Does your paper address subitem 1b-v?

Copy and paste relevant sections from the manuscript abstract (include quotes in quotation marks "like this" to indicate direct quotes from your manuscript), or elaborate on this item by providing additional information not in the ms, or briefly explain why the item is not applicable/relevant for your study

While the negative primary outcome of the trial (null findings on total-daily sedentary behavior change) was not the outcome of interest for this study, we stated adverse effects specific to this study in our abstract. In reporting these adverse effects, we stated: "the treadmill group favored prolonged standing bouts (30-60 min and >60 min)" and concluded that "Sit-to-stand desks exerted potentially more favorable physical behavior accumulation patterns than treadmill desks. Future active-workstation trials should consider strategies to promote more frequent long-term movement bouts and dissuade prolonged static postural fixity."

INTRODUCTION

2a) In INTRODUCTION: Scientific background and explanation of rationale

2a-i) Problem and the type of system/solution

Describe the problem and the type of system/solution that is object of the study: intended as stand-alone intervention vs. incorporated in broader health care program? Intended for a particular patient population? Goals of the intervention, e.g., being more cost-effective to other interventions, replace or complement other solutions? (Note: Details about the intervention are provided in “Methods” under 5)

	1	2	3	4	5	
subitem not at all important	○	○	○	○	○	essential

Does your paper address subitem 2a-i? *

Copy and paste relevant sections from the manuscript (include quotes in quotation marks "like this" to indicate direct quotes from your manuscript), or elaborate on this item by providing additional information not in the ms, or briefly explain why the item is not applicable/relevant for your study

The problem and proposed solution we tested in this trial are addressed with the following statements in the introduction:
i) " The workplace is considered to be a priority setting to reduce sedentary behavior [7] because a substantial proportion of the workforce is employed in full-time seated desk jobs [8-10]. Treadmill and sit-to-stand desks have gained popularity as alternatives to traditional seated workstations to promote interruptions in workplace sitting. A better understanding of how these desks alter the accumulation of physical behaviors among sedentary office workers may be useful to develop effective strategies to “move more and sit less” [6].”
ii) ii) " little experimental evidence exists on the impact of treadmill and sit-to-stand desks on the daily transitional balance between time spent in static postures (i.e., sitting and standing) versus stepping. Such evidence may be useful in tackling the public health challenge of promoting sitting less and moving more often in at risk populations.”

2a-ii) Scientific background, rationale: What is known about the (type of) system

Scientific background, rationale: What is known about the (type of) system that is the object of the study (be sure to discuss the use of similar systems for other conditions/diagnoses, if appropiate), motivation for the study, i.e. what are the reasons for and what is the context for this specific study, from which stakeholder viewpoint is the study performed, potential impact of findings [2]. Briefly justify the choice of the comparator.

	1	2	3	4	5	
subitem not at all important	○	○	○	○	○	essential

Does your paper address subitem 2a-ii? *

Copy and paste relevant sections from the manuscript (include quotes in quotation marks "like this" to indicate direct quotes from your manuscript), or elaborate on this item by providing additional information not in the ms, or briefly explain why the item is not applicable/relevant for your study

After setting up the background for the research question, our introduction addressed the rational for the study by stating that i) " little experimental evidence exists on the impact of treadmill and sit-to-stand desks on the daily transitional balance between time spent in static postures (i.e., sitting and standing) versus stepping.” and ii) "Most treadmill and sit-stand desk studies, to date, report effects on overall volume of time spent in waking physical behaviors with minimal information on the pattern of accumulating behavioral bouts."

2b) In INTRODUCTION: Specific objectives or hypotheses

Does your paper address CONSORT subitem 2b? *

Copy and paste relevant sections from the manuscript (include quotes in quotation marks "like this" to indicate direct quotes from your manuscript), or elaborate on this item by providing additional information not in the ms, or briefly explain why the item is not applicable/relevant for your study

Our introduction stated specific objectives of our study in the sub-section titled "Objectives of this Study".

METHODS

3a) Description of trial design (such as parallel, factorial) including allocation ratio

Does your paper address CONSORT subitem 3a? *

Copy and paste relevant sections from the manuscript (include quotes in quotation marks "like this" to indicate direct quotes from your manuscript), or elaborate on this item by providing additional information not in the ms, or briefly explain why the item is not applicable/relevant for your study

Subitem 3a was addressed in the methods of our study where we stated: "Data presented in this study is from a 12-month three-arm cluster-randomized controlled trial".

3b) Important changes to methods after trial commencement (such as eligibility criteria), with reasons

Does your paper address CONSORT subitem 3b? *

Copy and paste relevant sections from the manuscript (include quotes in quotation marks "like this" to indicate direct quotes from your manuscript), or elaborate on this item by providing additional information not in the ms, or briefly explain why the item is not applicable/relevant for your study

We had no protocol deviations to report.

3b-i) Bug fixes, Downtimes, Content Changes

Bug fixes, Downtimes, Content Changes: ehealth systems are often dynamic systems. A description of changes to methods therefore also includes important changes made on the intervention or comparator during the trial (e.g., major bug fixes or changes in the functionality or content) (5-iii) and other “unexpected events” that may have influenced study design such as staff changes, system failures/downtimes, etc. [2].

	1	2	3	4	5	
subitem not at all important	○	○	○	○	○	essential

Does your paper address subitem 3b-i?

Copy and paste relevant sections from the manuscript (include quotes in quotation marks "like this" to indicate direct quotes from your manuscript), or elaborate on this item by providing additional information not in the ms, or briefly explain why the item is not applicable/relevant for your study

Subitem 3b-i is not relevant to our study.

4a) Eligibility criteria for participants

Does your paper address CONSORT subitem 4a? *

Copy and paste relevant sections from the manuscript (include quotes in quotation marks "like this" to indicate direct quotes from your manuscript), or elaborate on this item by providing additional information not in the ms, or briefly explain why the item is not applicable/relevant for your study

Eligibility criteria were stated in the "Setting and Participants" subsection of our methods:

"Employees within these clusters were initially considered eligible if they were employed in a full-time seated-desk job, were between 18 and 65 years of age, had a body mass index greater than 25 kg/m2, did not engage in any structured physical activity on more than two days/week, and were free of limitations that prevented walking and standing in bouts lasting 40 to 60 min. An additional criterion for women was that they were not pregnant nor planning to become pregnant in the next year. Subjects were also screened for hypertension, diabetes, cardiovascular disease, and musculoskeletal conditions (i.e., joint, bone or muscle conditions) using a medical history questionnaire at baseline."

4a-i) Computer / Internet literacy

Computer / Internet literacy is often an implicit “de facto” eligibility criterion - this should be explicitly clarified.

	1	2	3	4	5	
subitem not at all important	○	○	○	○	○	essential

Does your paper address subitem 4a-i?

Copy and paste relevant sections from the manuscript (include quotes in quotation marks "like this" to indicate direct quotes from your manuscript), or elaborate on this item by providing additional information not in the ms, or briefly explain why the item is not applicable/relevant for your study

Subitem 4a-i is not relevant to our study.

4a-ii) Open vs. closed, web-based vs. face-to-face assessments:

Open vs. closed, web-based vs. face-to-face assessments: Mention how participants were recruited (online vs. offline), e.g., from an open access website or from a clinic, and clarify if this was a purely web-based trial, or there were face-to-face components (as part of the intervention or for assessment), i.e., to what degree got the study team to know the participant. In online-only trials, clarify if participants were quasi-anonymous and whether having multiple identities was possible or whether technical or logistical measures (e.g., cookies, email confirmation, phone calls) were used to detect/prevent these.

	1	2	3	4	5	
subitem not at all important	○	○	○	○	○	essential

Does your paper address subitem 4a-ii? *

Copy and paste relevant sections from the manuscript (include quotes in quotation marks "like this" to indicate direct quotes from your manuscript), or elaborate on this item by providing additional information not in the ms, or briefly explain why the item is not applicable/relevant for your study

Recruitment and enrollment specifications relevant to subitem 4a-ii were stated in the following sections of our supplementary "Detailed Protocol": Section III-b "Source of subjects and Recruitment methods", Section IV-a "Methods of enrollment, including procedures for patient registration and/or randomization", and section IV-b "Procedures for obtaining informed consent (including timing of consent process)".

In addition, in our manuscript we indicated the "face-to-face" components of our intervention relevant to this study by stating: "Enrolled individuals received initial face-to-face counseling sessions on the benefits of sitting less, standing and moving more. Supervisors of those participants in the treadmill and sit-to-stand desk groups received initial on-site training on the benefits of decreasing sedentary behavior in the workplace and on strategies to encourage participant engagement in the intervention, which they did so at regular department meetings. Participant counseling and supervisor trainings were repeated every three months for the treadmill and sit-to-stand desk groups, and these participants were also given feedback on their measured physical behaviors (described below) during these sessions.

“Finally, in section V-b of our supplementary "Detailed Protocol" we delineated measures of the trial that were performed on on-site face-to-face with study personnel versus at home by stating "The participant will complete measures 1 to 5 in a lab and measures 6 to 8 at home within 1-week of completing the former. Several of these measures will be recorded on a hard-copy data collection sheet."

4a-iii) Information giving during recruitment

Information given during recruitment. Specify how participants were briefed for recruitment and in the informed consent procedures (e.g., publish the informed consent documentation as appendix, see also item X26), as this information may have an effect on user self-selection, user expectation and may also bias results.

	1	2	3	4	5	
subitem not at all important	○	○	○	○	○	essential

Does your paper address subitem 4a-iii?

Copy and paste relevant sections from the manuscript (include quotes in quotation marks "like this" to indicate direct quotes from your manuscript), or elaborate on this item by providing additional information not in the ms, or briefly explain why the item is not applicable/relevant for your study

Subitem 4a-iii is addressed in our the supplementary "Detailed Protocol" under "b) Procedures for obtaining informed consent (including timing of consent process)".

4b) Settings and locations where the data were collected

Does your paper address CONSORT subitem 4b? *

Copy and paste relevant sections from the manuscript (include quotes in quotation marks "like this" to indicate direct quotes from your manuscript), or elaborate on this item by providing additional information not in the ms, or briefly explain why the item is not applicable/relevant for your study

We stated the setting of the intervention in the manuscript ("Sixty-six office-workers were recruited in clusters from The Massachusetts General Hospital and Northeastern University in Boston, MA") and described the details of the setting for recruitment in the "detailed protocol" supplement.

4b-i) Report if outcomes were (self-)assessed through online questionnaires

Clearly report if outcomes were (self-)assessed through online questionnaires (as common in web-based trials) or otherwise.

	1	2	3	4	5	
subitem not at all important	○	○	○	○	○	essential

Does your paper address subitem 4b-i? *

Copy and paste relevant sections from the manuscript (include quotes in quotation marks "like this" to indicate direct quotes from your manuscript), or elaborate on this item by providing additional information not in the ms, or briefly explain why the item is not applicable/relevant for your study

While the questionnaire-based self-assessments used in this trial were not relevant to this particular study, we stated in the "Detailed Protocol" that "items 6) Affect and stress, 7) Musculoskeletal discomfort symptoms, and 8) Dietary Intake were measured at home via questionnaire."

4b-ii) Report how institutional affiliations are displayed

Report how institutional affiliations are displayed to potential participants [on ehealth media], as affiliations with prestigious hospitals or universities may affect volunteer rates, use, and reactions with regards to an intervention.(Not a required item – describe only if this may bias results)

	1	2	3	4	5	
subitem not at all important	○	○	○	○	○	essential

Does your paper address subitem 4b-ii?

Copy and paste relevant sections from the manuscript (include quotes in quotation marks "like this" to indicate direct quotes from your manuscript), or elaborate on this item by providing additional information not in the ms, or briefly explain why the item is not applicable/relevant for your study

The authors have no conflicts of interest to disclose.

5) The interventions for each group with sufficient details to allow replication, including how and when they were actually administered

5-i) Mention names, credential, affiliations of the developers, sponsors, and owners

Mention names, credential, affiliations of the developers, sponsors, and owners [6] (if authors/evaluators are owners or developer of the software, this needs to be declared in a “Conflict of interest” section or mentioned elsewhere in the manuscript).

	1	2	3	4	5	
subitem not at all important	○	○	○	○	○	essential

Does your paper address subitem 5-i?

Copy and paste relevant sections from the manuscript (include quotes in quotation marks "like this" to indicate direct quotes from your manuscript), or elaborate on this item by providing additional information not in the ms, or briefly explain why the item is not applicable/relevant for your study

In our manuscript we mentioned the developers of the technology we used in our intervention by stating: "The height-adjustable desks used in this study were the WorkFit-D from Ergotron® Inc. For the treadmill desk group, a WorkFit-D desk was retrofitted with a treadmill (TR1200 DT-3, LifeSpan Fitness Inc.) for each participant."

5-ii) Describe the history/development process

Describe the history/development process of the application and previous formative evaluations (e.g., focus groups, usability testing), as these will have an impact on adoption/use rates and help with interpreting results.

	1	2	3	4	5	
subitem not at all important	○	○	○	○	○	essential

Does your paper address subitem 5-ii?

Copy and paste relevant sections from the manuscript (include quotes in quotation marks "like this" to indicate direct quotes from your manuscript), or elaborate on this item by providing additional information not in the ms, or briefly explain why the item is not applicable/relevant for your study

In our manuscript we described the source of instruction we provided participants on the proper use of the sit-to-stand and treadmill desks we installed where we stated:

"Prior to using the desks, participants were trained on appropriate ergonomic postures while standing and sitting at the height-adjustable desk and were instructed on gradually acclimating to standing and walking while working. These strategies were based on recommendations from the Occupational Safety and Health Administration [36, 37] and qualitative feedback from our prior formative work [38]."

5-iii) Revisions and updating

Revisions and updating. Clearly mention the date and/or version number of the application/intervention (and comparator, if applicable) evaluated, or describe whether the intervention underwent major changes during the evaluation process, or whether the development and/or content was “frozen” during the trial. Describe dynamic components such as news feeds or changing content which may have an impact on the replicability of the intervention (for unexpected events see item 3b).

	1	2	3	4	5	
subitem not at all important	○	○	○	○	○	essential

Does your paper address subitem 5-iii?

Copy and paste relevant sections from the manuscript (include quotes in quotation marks "like this" to indicate direct quotes from your manuscript), or elaborate on this item by providing additional information not in the ms, or briefly explain why the item is not applicable/relevant for your study

The trial was funded from National Institute for Occupational Safety and Health under contract award number 1R21OH010564-01. The intervention did not undergo major changes following approval from the funding agency and was carried out as originally designed in the application.

5-iv) Quality assurance methods

Provide information on quality assurance methods to ensure accuracy and quality of information provided [1], if applicable.

	1	2	3	4	5	
subitem not at all important	○	○	○	○	○	essential

Does your paper address subitem 5-iv?

Copy and paste relevant sections from the manuscript (include quotes in quotation marks "like this" to indicate direct quotes from your manuscript), or elaborate on this item by providing additional information not in the ms, or briefly explain why the item is not applicable/relevant for your study

Subitem 5-iv was addressed in section IX) "Monitoring and Quality Assurance" of our "Detailed Protocol" under subsections: "a) Independent monitoring of source data", "b) Safety monitoring", "c) Outcomes monitoring", and "d) Adverse event reporting guidelines".

5-v) Ensure replicability by publishing the source code, and/or providing screenshots/screen-capture video, and/or providing flowcharts of the algorithms used

Ensure replicability by publishing the source code, and/or providing screenshots/screen-capture video, and/or providing flowcharts of the algorithms used. Replicability (i.e., other researchers should in principle be able to replicate the study) is a hallmark of scientific reporting.

	1	2	3	4	5	
subitem not at all important	○	○	○	○	○	essential

Does your paper address subitem 5-v?

Copy and paste relevant sections from the manuscript (include quotes in quotation marks "like this" to indicate direct quotes from your manuscript), or elaborate on this item by providing additional information not in the ms, or briefly explain why the item is not applicable/relevant for your study

Subitem 5-v is not relevant to our study.

5-vi) Digital preservation

Digital preservation: Provide the URL of the application, but as the intervention is likely to change or disappear over the course of the years; also make sure the intervention is archived (Internet Archive, webcitation.org, and/or publishing the source code or screenshots/videos alongside the article). As pages behind login screens cannot be archived, consider creating demo pages which are accessible without login.

	1	2	3	4	5	
subitem not at all important	○	○	○	○	○	essential

Does your paper address subitem 5-vi?

Copy and paste relevant sections from the manuscript (include quotes in quotation marks "like this" to indicate direct quotes from your manuscript), or elaborate on this item by providing additional information not in the ms, or briefly explain why the item is not applicable/relevant for your study

Subitem 5-vi is not relevant to our study.

5-vii) Access

Access: Describe how participants accessed the application, in what setting/context, if they had to pay (or were paid) or not, whether they had to be a member of specific group. If known, describe how participants obtained “access to the platform and Internet” [1]. To ensure access for editors/reviewers/readers, consider to provide a “backdoor” login account or demo mode for reviewers/readers to explore the application (also important for archiving purposes, see vi).

	1	2	3	4	5	
subitem not at all important	○	○	○	○	○	essential

Does your paper address subitem 5-vii? *

Copy and paste relevant sections from the manuscript (include quotes in quotation marks "like this" to indicate direct quotes from your manuscript), or elaborate on this item by providing additional information not in the ms, or briefly explain why the item is not applicable/relevant for your study

Subitem 5-vii is not relevant to our study.

5-viii) Mode of delivery, features/functionalities/components of the intervention and comparator, and the theoretical framework

Describe mode of delivery, features/functionalities/components of the intervention and comparator, and the theoretical framework [6] used to design them (instructional strategy [1], behaviour change techniques, persuasive features, etc., see e.g., [7, 8] for terminology). This includes an in-depth description of the content (including where it is coming from and who developed it) [1]," whether [and how] it is tailored to individual circumstances and allows users to track their progress and receive feedback" [6]. This also includes a description of communication delivery channels and – if computer-mediated communication is a component – whether communication was synchronous or asynchronous [6]. It also includes information on presentation strategies [1], including page design principles, average amount of text on pages, presence of hyperlinks to other resources, etc. [1].

	1	2	3	4	5	
subitem not at all important	○	○	○	○	○	essential

Does your paper address subitem 5-viii? *

Copy and paste relevant sections from the manuscript (include quotes in quotation marks "like this" to indicate direct quotes from your manuscript), or elaborate on this item by providing additional information not in the ms, or briefly explain why the item is not applicable/relevant for your study

The theoretical framework of our trial was addressed in the methods section of our manuscript where we stated: "This was a multicomponent intervention to decrease sedentary behavior in the workplace and comprised of organizational-, environmental-, and individual-level strategies to target behavior change at both the individual and cluster levels [22]. "

In addition, we stated the how participant tracked their progress and received feedback when we stated: "Participant counseling and supervisor trainings were repeated every three months for the treadmill and sit-to-stand desk groups, and these participants were also given feedback on their measured physical behaviors (described below) during these sessions."

5-ix) Describe use parameters

Describe use parameters (e.g., intended “doses” and optimal timing for use). Clarify what instructions or recommendations were given to the user, e.g., regarding timing, frequency, heaviness of use, if any, or was the intervention used ad libitum.

	1	2	3	4	5	
subitem not at all important	○	○	○	○	○	essential

Does your paper address subitem 5-ix?

Copy and paste relevant sections from the manuscript (include quotes in quotation marks "like this" to indicate direct quotes from your manuscript), or elaborate on this item by providing additional information not in the ms, or briefly explain why the item is not applicable/relevant for your study

We stated the use parameters of the sit-to-stand and treadmill workstations in the methods section of our manuscript:

" Both treadmill and sit-to-stand desk workers were recommended to replace at least 3 hours of daily sedentary time with upright behaviors (i.e., three hours of standing/day in bouts of 10-30 min after acclimation for sit-to-stand desk participants, and two hours of walking/day and one hour of standing/day in bouts of 10-30 min after acclimation for treadmill desk participants). The prescribed duration of 10-30 min bouts were recommended to dissuade participants from engaging in prolonged postural fixity, as prolonged static standing has been suggested to result in unfavorable cardiovascular physiology [40, 41] that is similar to the detrimental effects observed after prolonged sitting for approximately >30 minutes of continuous sitting [28, 34, 35]. Seated-desk controls were recommended to engage in three 10-min bouts of moderate-vigorous intensity walking/day to meet the 2008 federal physical activity guidelines [42]."

5-x) Clarify the level of human involvement

Clarify the level of human involvement (care providers or health professionals, also technical assistance) in the e-intervention or as co-intervention (detail number and expertise of professionals involved, if any, as well as "type of assistance offered, the timing and frequency of the support, how it is initiated, and the medium by which the assistance is delivered". It may be necessary to distinguish between the level of human involvement required for the trial, and the level of human involvement required for a routine application outside of a RCT setting (discuss under item 21 – generalizability).

	1	2	3	4	5	
subitem not at all important	○	○	○	○	○	essential

Does your paper address subitem 5-x?

Copy and paste relevant sections from the manuscript (include quotes in quotation marks "like this" to indicate direct quotes from your manuscript), or elaborate on this item by providing additional information not in the ms, or briefly explain why the item is not applicable/relevant for your study

In our manuscript we described how participant in our treatment arm received organization support to reduce sedentary behavior when we stated: "Supervisors of those participants in the treadmill and sit-to-stand desk groups received initial on-site training on the benefits of decreasing sedentary behavior in the workplace and on strategies to encourage participant engagement in the intervention, which they did so at regular department meetings."

5-xi) Report any prompts/reminders used

Report any prompts/reminders used: Clarify if there were prompts (letters, emails, phone calls, SMS) to use the application, what triggered them, frequency etc. It may be necessary to distinguish between the level of prompts/reminders required for the trial, and the level of prompts/reminders for a routine application outside of a RCT setting (discuss under item 21 – generalizability).

	1	2	3	4	5	
subitem not at all important	○	○	○	○	○	essential

Does your paper address subitem 5-xi? *

Copy and paste relevant sections from the manuscript (include quotes in quotation marks "like this" to indicate direct quotes from your manuscript), or elaborate on this item by providing additional information not in the ms, or briefly explain why the item is not applicable/relevant for your study

Subitem 5-xi is not relevant to our study.

5-xii) Describe any co-interventions (incl. training/support)

Describe any co-interventions (incl. training/support): Clearly state any interventions that are provided in addition to the targeted eHealth intervention, as ehealth intervention may not be designed as stand-alone intervention. This includes training sessions and support [1]. It may be necessary to distinguish between the level of training required for the trial, and the level of training for a routine application outside of a RCT setting (discuss under item 21 – generalizability.

	1	2	3	4	5	
subitem not at all important	◯	◯	◯	◯	◯	essential

Does your paper address subitem 5-xii? *

Copy and paste relevant sections from the manuscript (include quotes in quotation marks "like this" to indicate direct quotes from your manuscript), or elaborate on this item by providing additional information not in the ms, or briefly explain why the item is not applicable/relevant for your study

We described training for organizational, individual and environmental components of our trial in the methods section where we stated:

"Enrolled individuals received initial face-to-face counseling sessions on the benefits of sitting less, standing and moving more. Supervisors of those participants in the treadmill and sit-to-stand desk groups received initial on-site training on the benefits of decreasing sedentary behavior in the workplace and on strategies to encourage participant engagement in the intervention, which they did so at regular department meetings. Participant counseling and supervisor trainings were repeated every three months for the treadmill and sit-to-stand desk groups, and these participants were also given feedback on their measured physical behaviors (described below) during these sessions.", and

"Prior to using the desks, participants were trained on appropriate ergonomic postures while standing and sitting at the height-adjustable desk and were instructed on gradually acclimating to standing and walking while working."

6a) Completely defined pre-specified primary and secondary outcome measures, including how and when they were assessed

Does your paper address CONSORT subitem 6a? *

Copy and paste relevant sections from the manuscript (include quotes in quotation marks "like this" to indicate direct quotes from your manuscript), or elaborate on this item by providing additional information not in the ms, or briefly explain why the item is not applicable/relevant for your study

Our study focused on secondary exploratory outcomes and we made this point in our manuscript when we stated: " The sample size of 66 equally distributed over three study arms was based on conservative power estimates of the change in the trial's primary outcome of total daily sedentary behavior accounting for a potential loss to follow-up. Details of our power and sample size estimation have been reported previously [22] and are also available in the supplemental "Detailed Protocol"., and "Since our outcomes are exploratory, we present post hoc determination of treatment-response trends for behavior change models indicated by unidirectional 95% confidence intervals, which do not overlap the null value. This exploratory approach avoids confirmatory statistical significance conclusions based on P values and thus, does not require the application of Bonferroni corrections. Such exploratory analyses are appropriate when the objective is to develop new hypotheses to further study observed phenomena [45]."

6a-i) Online questionnaires: describe if they were validated for online use and apply CHERRIES items to describe how the questionnaires were designed/deployed

If outcomes were obtained through online questionnaires, describe if they were validated for online use and apply CHERRIES items to describe how the questionnaires were designed/deployed [9].

	1	2	3	4	5	
subitem not at all important	○	○	○	○	○	essential

Does your paper address subitem 6a-i?

Copy and paste relevant sections from manuscript text

No online questionnaires were administered as part of the study presented in this manuscript.

6a-ii) Describe whether and how “use” (including intensity of use/dosage) was defined/measured/monitored

Describe whether and how “use” (including intensity of use/dosage) was defined/measured/monitored (logins, logfile analysis, etc.). Use/adoption metrics are important process outcomes that should be reported in any ehealth trial.

	1	2	3	4	5	
subitem not at all important	○	○	○	○	○	essential

Does your paper address subitem 6a-ii?

Copy and paste relevant sections from manuscript text

Sub item 6a-ii) was addressed in the manuscript methods and "detailed protocol" describing the activity monitor protocol used to measure resultant physical behaviors from work-station use and time in the workplace.

"Assessments included activity monitoring (activPALTM 3C, PAL Technologies Ltd., Glasgow, UK; 7 days on right thigh during waking hours) that occurred at baseline and after 3, 6 and 12 months. In our results we use the following abbreviations: M3 = month-3 follow-up, M6= month-6 follow-up, and M12= month-12 follow-up."

"Activity monitoring: This measurement will be conducted for a period of 1 week. Participants will be asked to wear 2 small activity monitors (53 x 35 x 7 mm, weight=15 grams), one on the right thigh and the other on the torso (chest or abdomen). The device will be attached to the skin using medical grade, hypoallergenic tape or soft elastic bands. We will measure time spent at work and outside the workplace for one week using radio frequency identification technology (RFID). A thin RFID sticker tag (120 mm x 44 mm x 0.4 mm) will be wrapped around the activity monitor on the torso. This will record every time a participant enters and leaves their place of work on an RFID reader installed in the building. During this 1-week period, participants will record the times they wore and removed the monitors and the times they arrived and left their workplace. Based on the data collected using these devices, participants will be provided with retrospective feedback every 3 months on their activity behavior. "

6a-iii) Describe whether, how, and when qualitative feedback from participants was obtained

Describe whether, how, and when qualitative feedback from participants was obtained (e.g., through emails, feedback forms, interviews, focus groups).

	1	2	3	4	5	
subitem not at all important	○	○	○	○	○	essential

Does your paper address subitem 6a-iii?

Copy and paste relevant sections from manuscript text

Qualitative feedback was not obtained from participants.

6b) Any changes to trial outcomes after the trial commenced, with reasons

Does your paper address CONSORT subitem 6b? *

Copy and paste relevant sections from the manuscript (include quotes in quotation marks "like this" to indicate direct quotes from your manuscript), or elaborate on this item by providing additional information not in the ms, or briefly explain why the item is not applicable/relevant for your study

There were no major outcome changes to the trial after commencement. Our exploration of physical behavior patterns is a post-study response to a new public health initiative to promote strategies for at risk populations to "move more and sit less", which the a priori "sedentary behavior" volume metric was unable to fully capture. Henceforth, sensor data collected for the primary objective was examined with greater granularity to answer exploratory research questions relevant to new public health guidelines not available at the time of the study.

7a) How sample size was determined

NPT: When applicable, details of whether and how the clustering by care provides or centers was addressed

7a-i) Describe whether and how expected attrition was taken into account when calculating the sample size

Describe whether and how expected attrition was taken into account when calculating the sample size.

	1	2	3	4	5	
subitem not at all important	○	○	○	○	○	essential

Does your paper address subitem 7a-i?

Copy and paste relevant sections from manuscript title (include quotes in quotation marks "like this" to indicate direct quotes from your manuscript), or elaborate on this item by providing additional information not in the ms, or briefly explain why the item is not applicable/relevant for your study

In the "detailed protocol" we stated:

"c) Power analyses
Sample size calculations are based on body weight change among participants (N=12) in our preliminary study.11 Our current study will have 22 participants in each of the three study arms. This will provide sufficient power to detect an effect size of 0.89 between each of the groups where the effect size = Δ/s (Δ: mean difference between two groups; s: common standard deviation). A standard deviation of 3.9 kg for weight observed in our pilot study corresponds to a mean detectable difference of 3.5 kg between groups. Although we anticipate minimal clustering within the departments at the study site, the power estimates presented above are based on an effective sample size of 60 subjects reflecting an intra-class correlation coefficient of 0.01. Comparisons between the most effective intervention and the control group will have substantially greater power reflecting larger differences in mean measurements."

7b) When applicable, explanation of any interim analyses and stopping guidelines

Does your paper address CONSORT subitem 7b? *

Copy and paste relevant sections from the manuscript (include quotes in quotation marks "like this" to indicate direct quotes from your manuscript), or elaborate on this item by providing additional information not in the ms, or briefly explain why the item is not applicable/relevant for your study

In the "detailed protocol" we specified stopping guidelines:

"b) Safety monitoring
A trained research staff member will meet with study participants twice a month and examine workstation function and set up. The staff member will also directly enquire if participants have any concerns regarding being part of the study. The participant can terminate participation in this study at any time if they do not wish to continue being a part of the study. There are no specific rules for stopping the study other than the occurrence of an adverse event."

8a) Method used to generate the random allocation sequence
NPT: When applicable, how care providers were allocated to each trial group

Does your paper address CONSORT subitem 8a? *

Copy and paste relevant sections from the manuscript (include quotes in quotation marks "like this" to indicate direct quotes from your manuscript), or elaborate on this item by providing additional information not in the ms, or briefly explain why the item is not applicable/relevant for your study

In the “detailed protocol” we stated:

"c) Treatment assignment and randomization
We will do a cluster randomization of various departments mentioned above into the control and 2 intervention groups. If employees from a department express interest in the study, the department will be cluster randomized into either the treadmill workstation, sit-stand workstation, or control group. Twenty-two volunteers will be randomly selected from each department group. In the event of insufficient volunteers in a group, the project manager will approach other departments within the hospital to solicit study participation."

8b) Type of randomisation; details of any restriction (such as blocking and block size)

Does your paper address CONSORT subitem 8b? *

Copy and paste relevant sections from the manuscript (include quotes in quotation marks "like this" to indicate direct quotes from your manuscript), or elaborate on this item by providing additional information not in the ms, or briefly explain why the item is not applicable/relevant for your study

Yes, we stated that our design was a “cluster-randomization”.

9) Mechanism used to implement the random allocation sequence (such as sequentially numbered containers), describing any steps taken to conceal the sequence until interventions were assigned

Does your paper address CONSORT subitem 9? *

Copy and paste relevant sections from the manuscript (include quotes in quotation marks "like this" to indicate direct quotes from your manuscript), or elaborate on this item by providing additional information not in the ms, or briefly explain why the item is not applicable/relevant for your study

Randomization followed informed consent, there was no blinding to treatment allocation prior to baseline assessments. We specified the sequence of randomization in the “detailed protocol”:

"If employees from a department express interest in the study, the department will be cluster randomized into either the treadmill workstation, sit-stand workstation, or control group after completing informed consent procedures."

10) Who generated the random allocation sequence, who enrolled participants, and who assigned participants to interventions

Does your paper address CONSORT subitem 10? *

Copy and paste relevant sections from the manuscript (include quotes in quotation marks "like this" to indicate direct quotes from your manuscript), or elaborate on this item by providing additional information not in the ms, or briefly explain why the item is not applicable/relevant for your study

Enrollment, randomization, and assignment to treatment arms were all carried out by the same study manager. We describe this process in the detailed protocol sections: "a) Methods of enrollment, including procedures for patient registration and/or randomization", "b) Procedures for obtaining informed consent (including timing of consent process)", and "c) Treatment assignment and randomization".

11a) If done, who was blinded after assignment to interventions (for example, participants, care providers, those assessing outcomes) and how

NPT: Whether or not administering co-interventions were blinded to group assignment

11a-i) Specify who was blinded, and who wasn't

Specify who was blinded, and who wasn't. Usually, in web-based trials it is not possible to blind the participants [1, 3] (this should be clearly acknowledged), but it may be possible to blind outcome assessors, those doing data analysis or those administering co-interventions (if any).

	1	2	3	4	5	
subitem not at all important	○	○	○	○	○	essential

Does your paper address subitem 11a-i? *

Copy and paste relevant sections from the manuscript (include quotes in quotation marks "like this" to indicate direct quotes from your manuscript), or elaborate on this item by providing additional information not in the ms, or briefly explain why the item is not applicable/relevant for your study

Subitem 11a-i) is not applicable to this study as this trial did not have a double-blind design.

11a-ii) Discuss e.g., whether participants knew which intervention was the "intervention of interest" and which one was the "comparator"

Informed consent procedures (4a-ii) can create biases and certain expectations - discuss e.g., whether participants knew which intervention was the "intervention of interest" and which one was the "comparator".

	1	2	3	4	5	
subitem not at all important	○	○	○	○	○	essential

Does your paper address subitem 11a-ii?

Copy and paste relevant sections from the manuscript (include quotes in quotation marks "like this" to indicate direct quotes from your manuscript), or elaborate on this item by providing additional information not in the ms, or briefly explain why the item is not applicable/relevant for your study

Participants were aware that the comparator was their usual "seated-desk" condition and that the interventions of interest were the "sit-to-stand" and "treadmill desks".

11b) If relevant, description of the similarity of interventions

(this item is usually not relevant for ehealth trials as it refers to similarity of a placebo or sham intervention to a active medication/intervention)

Does your paper address CONSORT subitem 11b? *

Copy and paste relevant sections from the manuscript (include quotes in quotation marks "like this" to indicate direct quotes from your manuscript), or elaborate on this item by providing additional information not in the ms, or briefly explain why the item is not applicable/relevant for your study

Subitem 11b is not relevant to our trial.

12a) Statistical methods used to compare groups for primary and secondary outcomes

NPT: When applicable, details of whether and how the clustering by care providers or centers was addressed

Does your paper address CONSORT subitem 12a? *

Copy and paste relevant sections from the manuscript (include quotes in quotation marks "like this" to indicate direct quotes from your manuscript), or elaborate on this item by providing additional information not in the ms, or briefly explain why the item is not applicable/relevant for your study

In our manuscript we stated: i) "Cluster effects for all outcomes were tested by calculating the intraclass correlation coefficients (ICCs) and their statistical significance (P <.05). " and ii) "The cluster effect did not significantly (all, P >.05) account for the variability in any of the outcome variables (Supplemental Table 2). "

12a-i) Imputation techniques to deal with attrition / missing values

Imputation techniques to deal with attrition / missing values: Not all participants will use the intervention/comparator as intended and attrition is typically high in ehealth trials. Specify how participants who did not use the application or dropped out from the trial were treated in the statistical analysis (a complete case analysis is strongly discouraged, and simple imputation techniques such as LOCF may also be problematic [4]).

	1	2	3	4	5	
subitem not at all important	O	O	O	O	O	essential

Does your paper address subitem 12a-i? *

Copy and paste relevant sections from the manuscript (include quotes in quotation marks "like this" to indicate direct quotes from your manuscript), or elaborate on this item by providing additional information not in the ms, or briefly explain why the item is not applicable/relevant for your study

In our manuscript we stated: " Losses to follow-up were handled as intent-to-treat and missing data attributable to unsystematic factors (e.g., monitor malfunction, sickness, improper device placement, forgetfulness to wear devices and/or log work hours, etc.) were imputed using joint multiple imputation [46, 47]."

12b) Methods for additional analyses, such as subgroup analyses and adjusted analyses

Does your paper address CONSORT subitem 12b? *

Copy and paste relevant sections from the manuscript (include quotes in quotation marks "like this" to indicate direct quotes from your manuscript), or elaborate on this item by providing additional information not in the ms, or briefly explain why the item is not applicable/relevant for your study

"Additionally, sensitivity analyses included participants who completed the one-year intervention [n's: total-day = 42 (13 controls, 13 sit-to-stand desks and 16 treadmill desks); workday = 35 (10 controls, 12 sit-to-stand desks and 13 treadmill desks)]. These analyses were conducted for all outcomes to determine if the overall treatment-response trends of the two interventions were altered when examining an ideal, but less conservative scenario of complete intervention compliance and to evaluate the sensitivity of the results to the handling of missing data and covariate adjustment [49]. "

X26) REB/IRB Approval and Ethical Considerations [recommended as subheading under "Methods"] (not a CONSORT item)

X26-i) Comment on ethics committee approval

	1	2	3	4	5	
subitem not at all important	○	○	○	○	○	essential

Does your paper address subitem X26-i?

Copy and paste relevant sections from the manuscript (include quotes in quotation marks "like this" to indicate direct quotes from your manuscript), or elaborate on this item by providing additional information not in the ms, or briefly explain why the item is not applicable/relevant for your study

Your answer **"Ethics approval was granted by Partner's HealthCare and Northeastern University institutional review boards in March of 2014, and all subjects provided written informed consent upon enrollment. "**

x26-ii) Outline informed consent procedures

Outline informed consent procedures e.g., if consent was obtained offline or online (how? Checkbox, etc.?), and what information was provided (see 4a-ii). See [6] for some items to be included in informed consent documents.

	1	2	3	4	5	
subitem not at all important	○	○	○	○	○	essential

Does your paper address subitem X26-ii?

Copy and paste relevant sections from the manuscript (include quotes in quotation marks "like this" to indicate direct quotes from your manuscript), or elaborate on this item by providing additional information not in the ms, or briefly explain why the item is not applicable/relevant for your study

We described these procedures in our "detailed protocol" under
"b) Procedures for obtaining informed consent (including timing of consent process)".

X26-iii) Safety and security procedures

Safety and security procedures, incl. privacy considerations, and any steps taken to reduce the likelihood or detection of harm (e.g., education and training, availability of a hotline)

	1	2	3	4	5	
subitem not at all important	○	○	○	○	○	essential

Does your paper address subitem X26-iii?

Copy and paste relevant sections from the manuscript (include quotes in quotation marks "like this" to indicate direct quotes from your manuscript), or elaborate on this item by providing additional information not in the ms, or briefly explain why the item is not applicable/relevant for your study

We described our workstation use safety protocols in the "detailed protocol" subsection "b) Safety monitoring".

RESULTS

13a) For each group, the numbers of participants who were randomly assigned, received intended treatment, and were analysed for the primary outcome

NPT: The number of care providers or centers performing the intervention in each group and the number of patients treated by each care provider in each center

Does your paper address CONSORT subitem 13a? *

Copy and paste relevant sections from the manuscript (include quotes in quotation marks "like this" to indicate direct quotes from your manuscript), or elaborate on this item by providing additional information not in the ms, or briefly explain why the item is not applicable/relevant for your study

"Sixty-six office workers were cluster-randomized into a seated-desk control (n = 21; 8 clusters), sit-to-stand desk (n = 23; 9 clusters), or treadmill desk (n = 22; 7 clusters) group." Supplemental Figures 1 and 2 show the N that were analyzed from these randomized groups at each follow-up point.

13b) For each group, losses and exclusions after randomisation, together with reasons

Does your paper address CONSORT subitem 13b? (NOTE: Preferably, this is shown in a CONSORT flow diagram) *

Copy and paste relevant sections from the manuscript (include quotes in quotation marks "like this" to indicate direct quotes from your manuscript), or elaborate on this item by providing additional information not in the ms, or briefly explain why the item is not applicable/relevant for your study

We presented consort flow diagrams in Supplemental Figures 1 and 2.

13b-i) Attrition diagram

Strongly recommended: An attrition diagram (e.g., proportion of participants still logging in or using the intervention/comparator in each group plotted over time, similar to a survival curve) or other figures or tables demonstrating usage/dose/engagement.

	1	2	3	4	5	
subitem not at all important	○	○	○	○	○	essential

Does your paper address subitem 13b-i?

Copy and paste relevant sections from the manuscript or cite the figure number if applicable (include quotes in quotation marks "like this" to indicate direct quotes from your manuscript), or elaborate on this item by providing additional information not in the ms, or briefly explain why the item is not applicable/relevant for your study

We presented attrition diagrams in Supplemental Figures 1 and 2.

14a) Dates defining the periods of recruitment and follow-up

Does your paper address CONSORT subitem 14a? *

Copy and paste relevant sections from the manuscript (include quotes in quotation marks "like this" to indicate direct quotes from your manuscript), or elaborate on this item by providing additional information not in the ms, or briefly explain why the item is not applicable/relevant for your study

In the methods section of our manuscript we stated: " Data analyzed in this study is from a 12-month three-arm cluster-randomized controlled trial titled 'Modifying the Workplace to Decrease Sedentary Behavior and Improve Health' (ClinicalTrials.gov ID No.: NCT02376504) conducted between March of 2014 and December 2016."

14a-i) Indicate if critical "secular events" fell into the study period

Indicate if critical "secular events" fell into the study period, e.g., significant changes in Internet resources available or "changes in computer hardware or Internet delivery resources"

	1	2	3	4	5	
subitem not at all important	◯	◯	◯	◯	◯	essential

Does your paper address subitem 14a-i?

Copy and paste relevant sections from the manuscript (include quotes in quotation marks "like this" to indicate direct quotes from your manuscript), or elaborate on this item by providing additional information not in the ms, or briefly explain why the item is not applicable/relevant for your study

Subitem 14a-i is not applicable to our study.

14b) Why the trial ended or was stopped (early)

Does your paper address CONSORT subitem 14b? *

Copy and paste relevant sections from the manuscript (include quotes in quotation marks "like this" to indicate direct quotes from your manuscript), or elaborate on this item by providing additional information not in the ms, or briefly explain why the item is not applicable/relevant for your study

The trial was carried out to completion as designed.

15) A table showing baseline demographic and clinical characteristics for each group

NPT: When applicable, a description of care providers (case volume, qualification, expertise, etc.) and centers (volume) in each group

Does your paper address CONSORT subitem 15? *

Copy and paste relevant sections from the manuscript (include quotes in quotation marks "like this" to indicate direct quotes from your manuscript), or elaborate on this item by providing additional information not in the ms, or briefly explain why the item is not applicable/relevant for your study

We present these details in our results:
" The control group comprised of 20 females and one male (eight African American/Black, 12 non-Hispanic Caucasians, and one Hispanic Caucasian; age= 41.9 ± 11.5 years; BMI= 33.3 ± 5.9 kg/m2). The sit-to-stand desk group comprised of 21 females and two males (two African American/Black, 16 non-Hispanic Caucasians, three Hispanic Caucasians, one Asian, and one other race/ethnicity; age= 43.6 ± 12.2 years; BMI= 30.8 ± 6.0 kg/m2). The treadmill desk group comprised of 18 females and four males (five African Americans/Black, 15 non-Hispanic Caucasians, one Hispanic Caucasian, and one other race/ethnicity; age= 50.4 ± 12.0 years; BMI= 33.5 ± 4.9 kg/m2). Self-reporting of medical history showed a prior or current history of hypertension in 11 participants (eight sit-to-stand desk, two treadmill desk and one control), diabetes in five participants (three sit-to-stand desk and two treadmill desk), cardiovascular disease in two participants (two treadmill desk), and musculoskeletal conditions in 24 participants (six sit-to-stand desk, nine treadmill desk and nine controls)."

15-i) Report demographics associated with digital divide issues

In ehealth trials it is particularly important to report demographics associated with digital divide issues, such as age, education, gender, social-economic status, computer/Internet/ehealth literacy of the participants, if known.

	1	2	3	4	5	
subitem not at all important	○	○	○	○	○	essential

Does your paper address subitem 15-i? *

Copy and paste relevant sections from the manuscript (include quotes in quotation marks "like this" to indicate direct quotes from your manuscript), or elaborate on this item by providing additional information not in the ms, or briefly explain why the item is not applicable/relevant for your study

Subitem 15-i is not applicable to our trial.

16) For each group, number of participants (denominator) included in each analysis and whether the analysis was by original assigned groups

16-i) Report multiple “denominators” and provide definitions

Report multiple “denominators” and provide definitions: Report N’s (and effect sizes) “across a range of study participation [and use] thresholds” [1], e.g., N exposed, N consented, N used more than x times, N used more than y weeks, N participants “used” the intervention/comparator at specific pre-defined time points of interest (in absolute and relative numbers per group). Always clearly define “use” of the intervention.

	1	2	3	4	5	
subitem not at all important	○	○	○	○	○	essential

Does your paper address subitem 16-i? *

Copy and paste relevant sections from the manuscript (include quotes in quotation marks "like this" to indicate direct quotes from your manuscript), or elaborate on this item by providing additional information not in the ms, or briefly explain why the item is not applicable/relevant for your study

In our manuscript we reported N's for the primary analyses and sensitivity analysis, which had a different N (i.e., complete-case analyses).

16-ii) Primary analysis should be intent-to-treat

Primary analysis should be intent-to-treat, secondary analyses could include comparing only "users", with the appropriate caveats that this is no longer a randomized sample (see 18-i).

	1	2	3	4	5	
subitem not at all important	○	○	○	○	○	essential

Does your paper address subitem 16-ii?

Copy and paste relevant sections from the manuscript (include quotes in quotation marks "like this" to indicate direct quotes from your manuscript), or elaborate on this item by providing additional information not in the ms, or briefly explain why the item is not applicable/relevant for your study

In our methods we stated: "Losses to follow-up were handled as intent-to-treat".

17a) For each primary and secondary outcome, results for each group, and the estimated effect size and its precision (such as 95% confidence interval)

Does your paper address CONSORT subitem 17a? *

Copy and paste relevant sections from the manuscript (include quotes in quotation marks "like this" to indicate direct quotes from your manuscript), or elaborate on this item by providing additional information not in the ms, or briefly explain why the item is not applicable/relevant for your study

We addressed this in the methods section of our manuscript where we stated:

"We present post hoc determination of treatment-response trends for behavior change models indicated by unidirectional 95% confidence intervals, which do not overlap the null value." and "Cohen's D effect sizes were calculated for all between and within group comparisons and categorized using standardized thresholds (i.e., 0.01= very small, 0.2= small, 0.5= medium, 0.8= large, 1.2= very large, and 2.0= huge) [48]. In our results we use the following abbreviations: VS= very small effect size, S= small effect size, M= medium effect size, L= large effect size, VL= very large effect size, and H= huge effect size. "

17a-i) Presentation of process outcomes such as metrics of use and intensity of use

In addition to primary/secondary (clinical) outcomes, the presentation of process outcomes such as metrics of use and intensity of use (dose, exposure) and their operational definitions is critical. This does not only refer to metrics of attrition (13-b) (often a binary variable), but also to more continuous exposure metrics such as "average session length". These must be accompanied by a technical description how a metric like a "session" is defined (e.g., timeout after idle time) [1] (report under item 6a).

	1	2	3	4	5	
subitem not at all important	○	○	○	○	○	essential

Does your paper address subitem 17a-i?

Copy and paste relevant sections from the manuscript (include quotes in quotation marks "like this" to indicate direct quotes from your manuscript), or elaborate on this item by providing additional information not in the ms, or briefly explain why the item is not applicable/relevant for your study

Item 17a-i is not applicable to our study.

17b) For binary outcomes, presentation of both absolute and relative effect sizes is recommended

Does your paper address CONSORT subitem 17b? *

Copy and paste relevant sections from the manuscript (include quotes in quotation marks "like this" to indicate direct quotes from your manuscript), or elaborate on this item by providing additional information not in the ms, or briefly explain why the item is not applicable/relevant for your study

We did not report binary outcomes.

18) Results of any other analyses performed, including subgroup analyses and adjusted analyses, distinguishing pre-specified from exploratory

Does your paper address CONSORT subitem 18? *

Copy and paste relevant sections from the manuscript (include quotes in quotation marks "like this" to indicate direct quotes from your manuscript), or elaborate on this item by providing additional information not in the ms, or briefly explain why the item is not applicable/relevant for your study

We presented a sensitivity analyses in the manuscript and described this in detail:

" Additionally, sensitivity analyses included participants who completed the one-year intervention [N's: total-day = 42 (13 controls, 13 sit-to-stand desks and 16 treadmill desks); workday = 35 (10 controls, 12 sit-to-stand desks and 13 treadmill desks)]. These analyses were conducted for all outcomes to determine if the overall treatment-response trends of the two interventions were altered when examining an ideal, but less conservative scenario of complete intervention compliance and to evaluate the sensitivity of the results to the handling of missing data and covariate adjustment [49]. "

18-i) Subgroup analysis of comparing only users

A subgroup analysis of comparing only users is not uncommon in ehealth trials, but if done, it must be stressed that this is a self-selected sample and no longer an unbiased sample from a randomized trial (see 16-iii).

	1	2	3	4	5	
subitem not at all important	○	○	○	○	○	essential

Does your paper address subitem 18-i?

Copy and paste relevant sections from the manuscript (include quotes in quotation marks "like this" to indicate direct quotes from your manuscript), or elaborate on this item by providing additional information not in the ms, or briefly explain why the item is not applicable/relevant for your study

We did not perform such sub group analysis.

19) All important harms or unintended effects in each group
(for specific guidance see CONSORT for harms)

Does your paper address CONSORT subitem 19? *

Copy and paste relevant sections from the manuscript (include quotes in quotation marks "like this" to indicate direct quotes from your manuscript), or elaborate on this item by providing additional information not in the ms, or briefly explain why the item is not applicable/relevant for your study

There were no adverse events to report as a consequence of the intervention.

19-i) Include privacy breaches, technical problems

Include privacy breaches, technical problems. This does not only include physical “harm” to participants, but also incidents such as perceived or real privacy breaches [1], technical problems, and other unexpected/unintended incidents. “Unintended effects” also includes unintended positive effects [2].

	1	2	3	4	5	
subitem not at all important	○	○	○	○	○	essential

Does your paper address subitem 19-i?

Copy and paste relevant sections from the manuscript (include quotes in quotation marks "like this" to indicate direct quotes from your manuscript), or elaborate on this item by providing additional information not in the ms, or briefly explain why the item is not applicable/relevant for your study

There were no privacy breaches nor technical problems to report. However, we do report the observed unintended effect of treadmill desk users choosing to stand for prolonged periods of time. We discussed this in the “Standing Bout Patterns” subsection of our manuscript’s discussion:

“Standing Bout Patterns

While the treadmill desk group engaged in more prescribed bouts (i.e., 10-30 min) relative controls after M6 and M12 both over the total-day and workday (~0.6-1.0 bout/day), an unintended response of engaging in more prolonged duration (i.e., >30 min) standing bouts was prominent among treadmill desk-users. Specifically, treadmill desk-users demonstrated trends of engaging in ~0.6-0.9 more prolonged standing bouts per day, relative to controls and sit-to-stand desk groups both over the total-day and workday after M3 and sustained after M12. On the contrary, sit-to-stand desk-users were more compliant with the intended intervention by increasing standing in prescribed bouts or shorter (i.e., 1-10 min) after M3 and sustained this after M12 both over the total-day (i.e., ~1-2 bouts/day) and workday (~3-8 bouts/day). These findings are noteworthy because prolonged static standing may be detrimental to cardiovascular health [39, 40]. We are unsure as to why users of treadmill-workstations preferred to engage in a much higher number of prolonged standing bouts as compared to the sit-to-stand desk group. Post-study interviews were unable to discern a cause for such behavior. While prolonged standing was not an issue in our sit-to-stand group, future active-workstation interventions should consider more frequent reinforcing messaging to dissuade prolonged standing bouts along with messaging about avoiding prolonged sitting amongst users of both desk types.”

19-ii) Include qualitative feedback from participants or observations from staff/researchers

Include qualitative feedback from participants or observations from staff/researchers, if available, on strengths and shortcomings of the application, especially if they point to unintended/unexpected effects or uses. This includes (if available) reasons for why people did or did not use the application as intended by the developers.

	1	2	3	4	5	
subitem not at all important	○	○	○	○	○	essential

Does your paper address subitem 19-ii?

Copy and paste relevant sections from the manuscript (include quotes in quotation marks "like this" to indicate direct quotes from your manuscript), or elaborate on this item by providing additional information not in the ms, or briefly explain why the item is not applicable/relevant for your study

While we did not conduct formal qualitative evaluations of the intervention from participants, we did communicate with participants to provide them with feedback on their progress every three months. We discussed anecdotal observations the researchers had from these face-to-face check-ins to speculate as to why treadmill desk users may have chosen to stand for prolonged periods of time. We stated:
“A possible explanation for the observed variability in the observed trends of sit-to-stand and treadmill desks on standing and stepping bout patterns may be the impact of the desk type on the ability to perform work. For example, when treadmill desk users were provided with feedback after M6 that they reversed positive stepping gains observed after M3, several participants in the treadmill-workstation group indicated a heavy work cycle between these two timepoints limited their ability to walk and work on the treadmill. Participants indicated that this particular work cycle required increased multitasking involving reading, writing, and typing. This reluctance to use the treadmill desk when performing office work that likely places a higher demand on cognitive function and involves multitasking coincides with prior evidence that treadmill desks may result in decreased performance of mouse proficiency, and typing and fine motor movement tasks [39, 51]. Another study showed that these interventions may result in minor declines in overall work performance within the first 3-5 months that may then return to and/or exceed baseline performance within a year of use [30]. It is likely that treadmill desk-users may have failed to outlast this temporary productivity adjustment period and chose to engage in prolonged standing bouts as a compensatory behavior to decreased use of the treadmill for walking after three months in order to maintain their work productivity. Given this learning curve, future active-workstation studies should consider the types of tasks office workers regularly engage in as barriers to replacing sedentary behavior with standing and stepping appropriately. Interventionists may need to design strategies to promote positive behavior change around such bouts of work during the first few months of commencing workstations use.”

DISCUSSION

22) Interpretation consistent with results, balancing benefits and harms, and considering other relevant evidence

NPT: In addition, take into account the choice of the comparator, lack of or partial blinding, and unequal expertise of care providers or centers in each group

22-i) Restate study questions and summarize the answers suggested by the data, starting with primary outcomes and process outcomes (use)

Restate study questions and summarize the answers suggested by the data, starting with primary outcomes and process outcomes (use).

	1	2	3	4	5	
subitem not at all important	○	○	○	○	○	essential

Does your paper address subitem 22-i? *

Copy and paste relevant sections from the manuscript (include quotes in quotation marks "like this" to indicate direct quotes from your manuscript), or elaborate on this item by providing additional information not in the ms, or briefly explain why the item is not applicable/relevant for your study

We discussed the comparisons between results of our trial and prior work in the "Sedentary Bout Patterns" subsection of our manuscript's discussion where we stated:
"Patterns of sedentary behavior bout accumulation observed in the sit-to-stand desk group were consistent with those reported in prior studies [27, 28] (i.e., sit-to-stand desk reducing prolonged sedentary bouts in relation to controls), but our study resulted in larger differences in usual sedentary bout duration relative to controls. Published findings from two cluster-randomized sit-to-stand desk trials with a follow-up of at least 12 months reported significant short- and long-term differences in workday time spent in prolonged bouts of sedentary behavior, which was defined as >30 min, favoring the intervention groups (i.e., 73 less min/workday after 3 months [28], and 35 and 45 less min/workday after 6 and 12 months, respectively in such bouts [27]). This is consistent with our finding that sit-to-stand desks reduced the accumulation of prolonged sedentary bouts. One of these studies also reported significant short-term differences in the duration of workday usual sedentary bouts in sit-to-stand desk users relative to controls (i.e., -4.4 min) [28], which is consistent with but shorter than the duration found in our study, which was -20.3 min.
Our study is the first to report the impact of treadmill desks on both the distribution of sedentary bouts across temporal categories and usual sedentary bout duration, as well as a head-to-head comparison of such metrics with sit-to-stand desks."

22-ii) Highlight unanswered new questions, suggest future research

Highlight unanswered new questions, suggest future research.

	1	2	3	4	5	
subitem not at all important	○	○	○	○	○	essential

Does your paper address subitem 22-ii?

Copy and paste relevant sections from the manuscript (include quotes in quotation marks "like this" to indicate direct quotes from your manuscript), or elaborate on this item by providing additional information not in the ms, or briefly explain why the item is not applicable/relevant for your study

Subitem 22-ii was addressed in the conclusions section of the manuscript's discussion.

20) Trial limitations, addressing sources of potential bias, imprecision, and, if relevant, multiplicity of analyses

20-i) Typical limitations in ehealth trials

Typical limitations in ehealth trials: Participants in ehealth trials are rarely blinded. Ehealth trials often look at a multiplicity of outcomes, increasing risk for a Type I error. Discuss biases due to non-use of the intervention/usability issues, biases through informed consent procedures, unexpected events.

	1	2	3	4	5	
subitem not at all important	○	○	○	○	○	essential

Does your paper address subitem 20-i? *

Copy and paste relevant sections from the manuscript (include quotes in quotation marks "like this" to indicate direct quotes from your manuscript), or elaborate on this item by providing additional information not in the ms, or briefly explain why the item is not applicable/relevant for your study

We discussed both strengths and limitations of our study in a the final subsection of the discussion.

21) Generalisability (external validity, applicability) of the trial findings

NPT: External validity of the trial findings according to the intervention, comparators, patients, and care providers or centers involved in the trial

21-i) Generalizability to other populations

Generalizability to other populations: In particular, discuss generalizability to a general Internet population, outside of a RCT setting, and general patient population, including applicability of the study results for other organizations

	1	2	3	4	5	
subitem not at all important	○	○	○	○	○	essential

Does your paper address subitem 21-i?

Copy and paste relevant sections from the manuscript (include quotes in quotation marks "like this" to indicate direct quotes from your manuscript), or elaborate on this item by providing additional information not in the ms, or briefly explain why the item is not applicable/relevant for your study

We addressed the generalizability and limitations thereof in the "Strength and Limitations" subsection of our manuscript's discussion.

21-ii) Discuss if there were elements in the RCT that would be different in a routine application setting

Discuss if there were elements in the RCT that would be different in a routine application setting (e.g., prompts/reminders, more human involvement, training sessions or other co-interventions) and what impact the omission of these elements could have on use, adoption, or outcomes if the intervention is applied outside of a RCT setting.

	1	2	3	4	5	
subitem not at all important	○	○	○	○	○	essential

Does your paper address subitem 21-ii?

Copy and paste relevant sections from the manuscript (include quotes in quotation marks "like this" to indicate direct quotes from your manuscript), or elaborate on this item by providing additional information not in the ms, or briefly explain why the item is not applicable/relevant for your study

Given the exploratory nature of our study, we chose not to speculate on how the elements of our RCT would be different in a routine application study. Before such a recommendation can be made, more research is warranted with larger trials powered to statistically confirm the treatment responses observed in our trial.

OTHER INFORMATION

23) Registration number and name of trial registry

Does your paper address CONSORT subitem 23? *

Copy and paste relevant sections from the manuscript (include quotes in quotation marks "like this" to indicate direct quotes from your manuscript), or elaborate on this item by providing additional information not in the ms, or briefly explain why the item is not applicable/relevant for your study

We included this information in the “Acknowledgements” subsection at the end of the manuscript.
“This trial is registered in ClinicalTrials.gov under the identifier number NCT02376504 and title, “Modifying the workplace to decrease sedentary behavior and improve health.”

24) Where the full trial protocol can be accessed, if available

Does your paper address CONSORT subitem 24? *

Cite a Multimedia Appendix, other reference, or copy and paste relevant sections from the manuscript (include quotes in quotation marks "like this" to indicate direct quotes from your manuscript), or elaborate on this item by providing additional information not in the ms, or briefly explain why the item is not applicable/relevant for your study

The full protocol was referenced in our Methods and is available as a supplement:
“The full protocol detailing all the measures used in the study as well as enrollment and attrition figures are available in supplementary material (i.e., see “Detailed Protocol” and Supplemental Figures 1 and 2).”

25) Sources of funding and other support (such as supply of drugs), role of funders

Does your paper address CONSORT subitem 25? *

Copy and paste relevant sections from the manuscript (include quotes in quotation marks "like this" to indicate direct quotes from your manuscript), or elaborate on this item by providing additional information not in the ms, or briefly explain why the item is not applicable/relevant for your study

We included this information in the “Acknowledgements” subsection at the end of the manuscript.
“This work was enabled by funding from the National Institute for Occupational Safety and Health under contract award number 1R21OH010564-01. The project was also supported by Grant Number 1UL1TR001102.”

X27) Conflicts of Interest (not a CONSORT item)

X27-i) State the relation of the study team towards the system being evaluated

In addition to the usual declaration of interests (financial or otherwise), also state the relation of the study team towards the system being evaluated, i.e., state if the authors/evaluators are distinct from or identical with the developers/sponsors of the intervention.

	1	2	3	4	5	
subitem not at all important	○	○	○	○	○	essential

Does your paper address subitem X27-i?

Copy and paste relevant sections from the manuscript (include quotes in quotation marks "like this" to indicate direct quotes from your manuscript), or elaborate on this item by providing additional information not in the ms, or briefly explain why the item is not applicable/relevant for your study

We reported no conflicts of interest at the end of our manuscript:
“Conflicts of Interest
The authors report no conflict of interest. The results of the study are presented clearly, honestly, and without fabrication, falsification, or inappropriate data manipulation.”

About the CONSORT EHEALTH checklist

As a result of using this checklist, did you make changes in your manuscript? *

- ○ yes, major changes
- ◉ yes, minor changes
- ○ no

What were the most important changes you made as a result of using this checklist?

Title and abstract detail modifications and inclusion of a full protocol document in our supplementary material.

How much time did you spend on going through the checklist INCLUDING making changes in your manuscript *

Approximately eight to ten hours.

As a result of using this checklist, do you think your manuscript has improved? *

- (selected) yes
- no
- Other:

Would you like to become involved in the CONSORT EHEALTH group?

This would involve for example becoming involved in participating in a workshop and writing an "Explanation and Elaboration" document

- yes
- (selected) no
- Other:

Clear selection

Any other comments or questions on CONSORT EHEALTH

No

Your answer must have a minimum of 25 characters.

STOP - Save this form as PDF before you click submit

To generate a record that you filled in this form, we recommend to generate a PDF of this page (on a Mac, simply select "print" and then select "print as PDF") before you submit it.

When you submit your (revised) paper to JMIR, please upload the PDF as supplementary file.

Don't worry if some text in the textboxes is cut off, as we still have the complete information in our database. Thank you!

Final step: Click submit !

Click submit so we have your answers in our database!

Submit Clear form

Never submit passwords through Google Forms.

This content is neither created nor endorsed by Google. Report Abuse - Terms of Service - Privacy Policy

Google Forms
